# Integrating Social Assistive Robots, IoT, Virtual Communities and Smart Objects to Assist at-Home Independently Living Elders: the MoveCare Project

**DOI:** 10.1007/s12369-021-00843-0

**Published:** 2022-02-14

**Authors:** Matteo Luperto, Javier Monroy, Jennifer Renoux, Francesca Lunardini, Nicola Basilico, Maria Bulgheroni, Angelo Cangelosi, Matteo Cesari, Manuel Cid, Aladar Ianes, Javier Gonzalez-Jimenez, Anastasis Kounoudes, David Mari, Victor Prisacariu, Arso Savanovic, Simona Ferrante, N. Alberto Borghese

**Affiliations:** 1 Applied Intelligent System Lab, Department of Computer Science, University of Milan, Milano, Italy; 2 Machine Perception and Intelligent Robotics Group (MAPIR), Department of System Engineering and Automation, University of Malaga, and Instituto de Investigación Biomédica de Málaga–IBIMA, Malaga, Spain; 3 Machine Perception and Interaction Lab, Örebro University, Örebro, Sweden; 4 NearLab, Department of Electronics, Information and Bioengineering, Politecnico di Milano, Milano, Italy; 5 AB.Acus SRL, Milan, Italy; 6 University of Manchester, Manchester, UK; 7 IRCCS Istituti Clinici Scientifici Maugeri, University of Milan, Milan, Italy; 8 Junta de Extremadura, Merida, Spain; 9 Korian, Milan, Italy; 10 SignalGeneriX, Limassol, Cyprus; 11 EURECAT, Barcelona, Spain; 12 University of Oxford, Oxford, UK; 13 Smart Com, Ljubjana, Slovenia

**Keywords:** Socially assistive robots, IoT network, Ambient Assisted Living, Monitoring, Virtual communities

## Abstract

The integration of Ambient Assisted Living (AAL) frameworks with Socially Assistive Robots (SARs) has proven useful for monitoring and assisting older adults in their own home. However, the difficulties associated with long-term deployments in real-world complex environments are still highly under-explored. In this work, we first present the MoveCare system, an unobtrusive platform that, through the integration of a SAR into an AAL framework, aimed to monitor, assist and provide social, cognitive, and physical stimulation in the own houses of elders living alone and at risk of falling into frailty. We then focus on the evaluation and analysis of a long-term pilot campaign of more than 300 weeks of usages. We evaluated the system’s acceptability and feasibility through various questionnaires and empirically assessed the impact of the presence of an assistive robot by deploying the system *with* and *without* it. Our results provide strong empirical evidence that Socially Assistive Robots integrated with monitoring and stimulation platforms can be successfully used for long-term support to older adults. We describe how the robot’s presence significantly incentivised the use of the system, but slightly lowered the system’s overall acceptability. Finally, we emphasise that real-world long-term deployment of SARs introduces a significant technical, organisational, and logistical overhead that should not be neglected nor underestimated in the pursuit of long-term robust systems. We hope that the findings and lessons learned from our work can bring value towards future long-term real-world and widespread use of SARs.

## Introduction

“Senectus ipsa est morbus” stated the Latin playwright Terentius in the first century BC. Indeed, counteracting elders’ decline is today among the top priorities of national health and social security institutions in all developed countries, where the population is ageing: low birth rates and higher life expectancy are changing the shape of the age pyramid, with a steep transition towards a much older population. Ageing is followed by a physical and cognitive decline that already has an enormous social and economic impact on society. This impact is worsened by the changes in the typical family structure [44], for which the number of elders living alone has largely increased [2].

Several research programs and projects have been recently financed at both national and European levels to address such an issue, including AAL-Europe^1^ Pharaon^2^, or SmartBEAR^3^, among the most notable ones. The mainstream approach represented by these research efforts concentrates on deploying wearable solutions, requiring the elder to carry specific devices to provide monitoring and assistance.

Against this background, Socially Assistive Robots (SARs) have a potentially prominent role, as they could embody intelligent and adaptive service providers for elders at home. However, and despite the promising results shown by some early works when assessing the capabilities of such platforms, the use of SARs is still in its early stages as these systems are often perceived as providers only of a limited set of functionalities [18]. That is why recent projects like EnrichMe [59], SYMPARTNER [27], Robot-Era [8], or GiraffPlus [11], have proposed the integration of SARs with Ambient Assisted Living (AAL) environments, aiming to increase the set of capabilities and functionalities that SARs offer to their users, and improve their level of assistance.

One central issue is that, despite the experimental results of many of such studies that clearly encourage deployments for long periods, a proper assessment of such a statement still calls for deep and sustained on-the-field validation [33]. Two main open challenges well represent this requirement. The first is studying their *technical* and *functional feasibility*, to identify the most critical factors that might impact this domain. The second concerns their *acceptability*, a feature that, when assessed over weeks or even months, might reveal strengths and weaknesses largely unobserved by many short-term campaigns presented in the literature. These two challenges are intertwined.

In this work, we focus and provide extensive results on the latter problem: long-term acceptability. Moreover, we also draw practical insights on how to devise a feasible, on-the-field deployment that can sustain fully autonomous operations for an extended time. To do so, we introduce a heterogeneous SAR-based platform with advanced functionalities that enables long-term monitoring, assistance, and social, cognitive, and physical stimulation towards an active life as required by the elder population itself [12]. This platform, named MoveCare (Multiple-actOrs Virtual Empathic CARegiver for the Elder^4^ [37]), integrates different technologies in a completely unobtrusive way: an Internet-of-Things (IoT) sub-system, specific smart objects, and a Community-Based Activity Centre (CBAC), all coordinated by an intelligent Virtual Caregiver (VC) embodied in a socially assistive robot.

The system was tested in an extensive pilot experimental campaign by deploying it to operate autonomously at elders’ homes for at least 10 continuous weeks, collecting a total of more than 300 weeks of usage data. The evaluation of the system was performed using structured questionnaires and by analysing such collected data. Moreover, to assess the long-term acceptability and feasibility with respect to a baseline, only half of the users tested the entire platform *with* the robot; the other half were provided only with all the other components. This allowed us to compare the two conditions between-subjects, from which to assess the impact of the robot’s role in the system.

In summary, the contributions of this work are the following:

- we present a SAR-based AAL framework that, by integrating several components, enables assistance, monitoring, and social, cognitive, and physical stimulation to older adults living alone;
- we assess the long-term acceptability and feasibility of such a system by discussing the results obtained in its long-term deployment in the own house of end-users;
- we provide an in-depth comparison of the data from users with and without the robotic platform, analyzing the pros and cons of integrating a mobile platform within an AAL framework.

Preliminary versions of this work have been presented in [40, 46, 55].

## Related Work

When surveying the contributions among SARs, the authors of [1] distinguished between *service robots*, aiming at helping users in daily activities, and *companion robots*, as [63], targeting the psychological well-being of their owners. Our work focuses on the first category, service robots, which currently present two significant drawbacks: (1) they often offer simple functionalities, mainly associated to monitoring, and the gap between these functionalities and the ones required by end-users still consistent; (2) deployment of such robots in real-world working conditions is yet in early stages, and there is little evidence obtained from actual long-term deployment in uncontrolled environments [48]. In this section, we will review various pieces of work that have addressed any of these two drawbacks and analyse how these studies differ from the approach we take in this work.

To identify which services older adults expect from service robots, previous works used structured interviews that were performed by asking a set of questions to older adults, often after a controlled demonstration, to show examples of the robot’s capabilities [23, 53]. Other pieces of work conducted interviews in focus groups by directly asking the potential users to describe the functionalities they would expect from such platforms [12, 51, 65]. This second approach exploited demonstrations too, typically with limited autonomy, such as teleoperation or semi-autonomous Wizard-of-Oz (WOZ) design [18, 31]. Only a limited set of such identified functionalities have been implemented in SARs, such as fall detection [3], meal assistance [29], or information and stimulation through messages [17].

Integration of SARs with AAL environments has been proposed to improve the robots’ capabilities and allow for more general service robots [7, 54]. For instance, the GiraffPlus project [11] deployed a teleoperated mobile robot to the elder’s home, together with a network of sensors, to monitor daily activities. In this project, however, the robot is semi-autonomous, meaning that an external user controls it to navigate the elder’s house when needed, and the system eases navigation. Integration of autonomous robots with AAL platforms is studied in [4, 18, 24] with robots whose primarily goal is to identify possible falls. Their integration with a broader AAL architecture offers additional services as reminders, pick and place of objects, and suggestions to perform entertainment activities.

One recent example is the Robot-Era project [8, 15], which investigated the technical feasibility, acceptance and satisfaction of older adults when using several functionalities provided by three different robots dedicated respectively to domestic, condominium, and outdoor environments. Elders were allowed to test the functionalities of autonomous SARs by performing with them a set of scenarios selected from those offered by the robot. Interestingly, older adults were involved during the entire development phase of the robots and the scenarios in a continuous integration framework. The evaluation was performed in a challenging but controlled environment: a sensorized living-lab apartment [8], and for a limited amount of time. This project focused on showing the feasibility of the integration of AAL with SARs and its potential applications (e.g., with live controlled demonstrations), while leaving open the challenge of studying a use case for actual real-world implementation. However, the evaluation of robotic systems in real settings is fundamental to discover challenges posed by such environments [27, 33] and fill the gap towards widespread adoption. The urge of tackling this task is widely recognised and critical to enable effective long-term deployments [48].

Nonetheless, only a few studies have deployed SARs for real-world evaluation in conditions similar to widespread deployment, as we do in this work. A relevant example is Strands [30], where an autonomous social robot was deployed for several weeks in the common areas of assisted living facilities. Unlike the work presented in this paper, the robot from Strands was deployed in large-scale environments to assist multiple users simultaneously (e.g., by giving directions). Such a context poses different challenges than those assessed in our work, where the goal is to provide fine-grained assistance and monitoring of a single user in their own house.

A SAR similar to ours can be found in the projects CompanionAble and SERROGA [26, 28], which presented performance results of long-term tests in private apartments, similar to those obtained in our pilot study. More recently, the project SYMPARTNER [27] showed the results obtained in a 20-weeks field study with 20 elders (1 week for each participant where the system was available to the user for 4 days). Compared to this series of studies, our work investigated a much longer interaction between elders and robots, where each robot is, moreover, deployed within a broader integrated framework of several components offering multiple functionalities to elders.

**Fig. 1:**
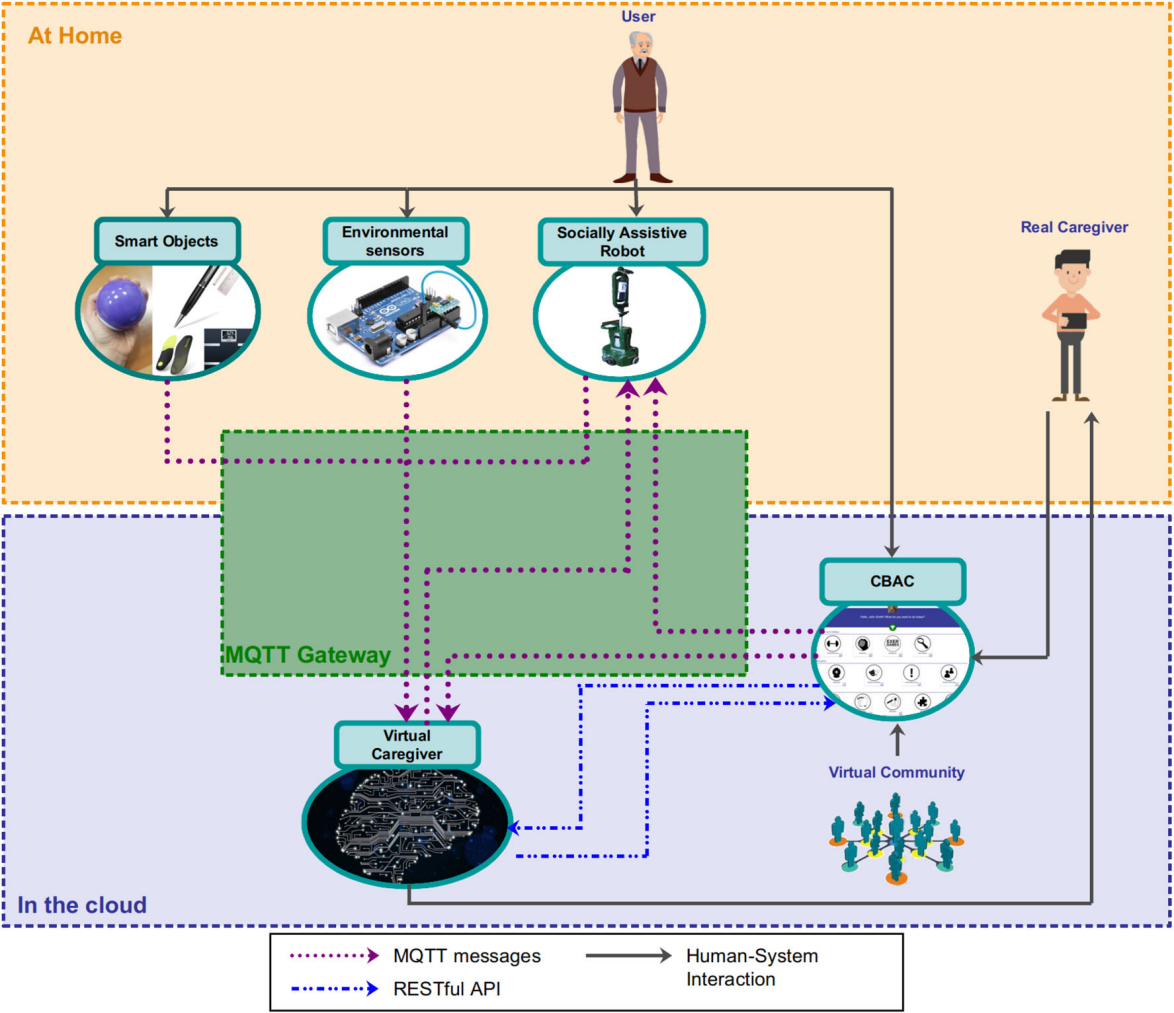
High-level overview of the MoveCare system. The system is composed of three components installed in the user’s home (the smart objects, the environmental sensors, and the service robot) and two components deployed in the cloud (the Virtual Caregiver and the Community-Based Activity Centre (CBAC)). Most of the communication between the components is performed through an MQTT Gateway. In addition, some communication between components in the cloud is performed through RESTful APIs. The users and human caregivers interact with the system through various interfaces

**Fig. 2:**
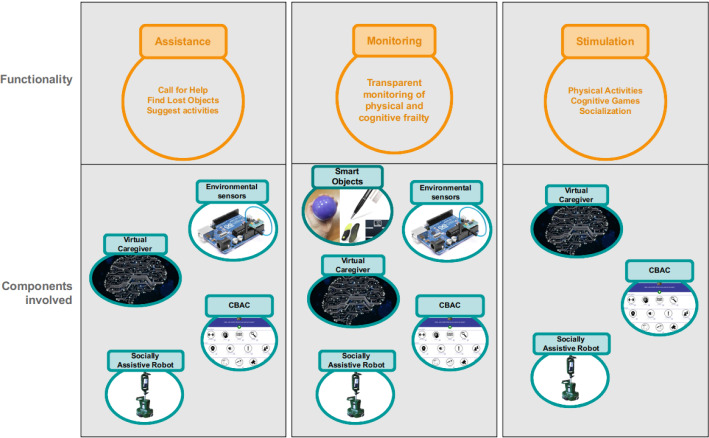
The functionalities implemented in the MoveCare platform with the components involved in their realisation

Similar to our work, the EnrichMe project [59] assessed the feasibility of long-term deployments inside the house of 10 elders for 10 weeks. The main objective of this project was to provide everyday-use tools and applications to assist the elderly user at home. These tools focused on health monitoring (body temperature, heart rate, and breathing), complementary care (diet and medicine reminder, physical and cognitive exercises), and everyday support (phone calls, object search, weather and news provider). While both the EnrichMe and the MoveCare projects present similarities in their platform and deployment, they differ by their focus and, therefore, the type of scenarios they support. The EnrichMe project focused on assisting the elder in their everyday tasks, while our work focused on monitoring early mild cognitive impairment and stimulating the users physically, cognitively, and socially through dedicated applications. Therefore, we included social activities and smart objects to detect frailty indicators in addition to environmental sensors. We also included the elder’s human caregiver in the loop.

To the best of our knowledge, the integration of assistive robots with monitoring frameworks to provide effective long-term interventions in the physical, cognitive, and most importantly, social domain, while also investigating the possibility to perform early detection of early signs of frailty in the long-term, has not been investigated so far. Previous work considered the use of smart-home monitoring [13, 56], and the detection of signs of frailty [60], but without the integration with a robotic platform nor the personalised interventions that our work considers.

## The MoveCare Platform

The primary goal of the MoveCare platform is to monitor, support, assist and stimulate pre-frail older adults [10] who live alone. The term “frailty” encompasses a set of vulnerabilities typically conveyed by a cognitive and physical decline in older adults. These vulnerabilities concur in amplifying the risks of major diseases, hindering independent living capability, and increasing the need for assisted living services or nursing homes. Our platform has been designed to meet the needs of both these elders and their caregivers.

Such needs have been elicited through an in-depth investigation utilizing questionnaires, interviews, and focus groups [12]. The different indications that were collected in such an activity have been translated into a set of functionalities. From these, an adequate set of components that, working together, could implement them has been selected. Such components are integrated into a single platform, presented in Fig. 1, in which a virtual caregiver orchestrates their operation. Particular care has been devoted to designing interfaces that would make the system easy to use for people with limited experience and proficiency with technology. Moreover, the system has been designed to be minimally invasive: it neither requires that the elders change their habits nor wear any particular device that would make the system of little usability, especially in the long run. Finally, no other modification in the elder’s home, besides the placement of environmental sensors, should be put in place for proper deployment of the system.

### The MoveCare Functionalities

An overview of the MoveCare functionalities is shown in Fig. 2. A critical functionality required for any AAL system is that of providing safety to its user. Elders should always be able to call for help and be sure that someone will respond to their help requests, especially when they are home alone.

A second key functionality is monitoring the physical and cognitive state to early detect possible decline. Three main approaches can be identified [32]: the use of the digital version of classical clinical tests (normally carried out with paper and pencil), the development of novel tests explicitly designed for mobile platforms, and the analysis of new data streams. The latter is the most innovative as it can be implemented in a completely unobtrusive way. All these three approaches have been integrated inside the MoveCare platform.

One last functionality meets a practical need often encountered in elders’ everyday life: help in finding lost objects (e.g., keys, wallet). The service robot is the key actor in providing this aid, which proved to play an essential role in increasing trust in the platform.

Our system presents a proof of concept of how all such functionalities can be effectively integrated into a single framework for a long period of time and how the robot can be an enabling factor for that. A deeper analysis of the results and impact of such functionalities (e.g., validation and longitudinal analysis of monitoring data, evaluation of the effect of stimulation) are beyond the scope of this work.

As mentioned previously, the human caregivers are part of the system and act as the primary contact point in case of a problem: they are notified if the users call for help, and interact with them through the robot. Moreover, the caregivers can participate in social activities with older adults using the CBAC.

**Table 1:**
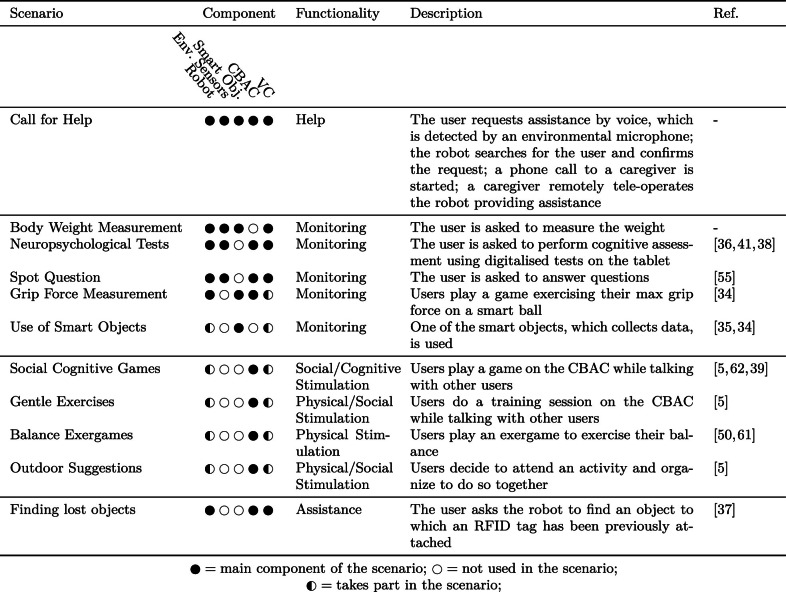
A list of the scenarios proposed by the MoveCare framework, describing the functionality they address and the components involved. A full description of the scenarios is provided in Appendix 10

### The MoveCare Scenarios

The functionalities introduced above have been framed into a set of *scenario*s that describe how the elders and caregivers can use the MoveCare platform. Scenarios have been designed focusing on their socio-clinical impact, the user’s interest in them, and their technical feasibility. In each scenario, the user interacts with one or more of the MoveCare components. A scenario can be started both by the user (e.g., the Call for Help scenario is started when the user requests help from the system) or by the system itself (e.g., the system asks the user to perform a body-weight measurement for monitoring purposes). A list of the available scenarios, describing the components and functionalities involved, is reported in Table 1, and a complete description of each scenario is given in Table 10, in the Appendix. As the MoveCare system is provided to users in two configurations, with and without the robot, we described in the appendix how scenarios are adapted when the robot is not present. It must be noticed that, as a natural consequence of not having the mobile robot, the without-robot configuration provides a reduced set of the described scenarios.

## Main MoveCare Components

The MoveCare system is constituted of the five main components depicted in Fig. 1. Three of these components are installed in the home of the user living alone and independently, while the other two components are deployed in the cloud:

- **Giraff-X** (Sect. 4.1), a Socially Assistive Robot (SAR) with advanced sensing capabilities.
- **Environmental Sensors** (Sect. 4.2), a set of domotic sensors integrated inside an IoT network that collects data about the user’s daily living activities and stores them in the cloud. The data is then available to the other system’s components.
- **Smart Objects** (Sect. 4.3), a set of specifically designed objects to perform unobtrusive monitoring and assistance.
- **Community-Based Activity Center** (CBAC, Sect. 4.4), a web platform providing cognitive, physical, and social activities, accessed through a tablet or a TV set-top box.
- **Virtual Caregiver** (Sect. 4.5), a virtual assistant providing intelligent supervision to the whole platform by reasoning on the collected data, determining the type and number of needed interventions, and managing their execution.

These components work together to perform the scenarios described in Table 1. Hereafter, we show how, by combining and coordinating these different components, the previously described scenarios have been realised, seeking an unobtrusive and acceptable setup. For a detailed list of components taking part in the proposed system, see Table 9, in the Appendix.

### The Socially Assistive Mobile Robot

**Fig. 3:**
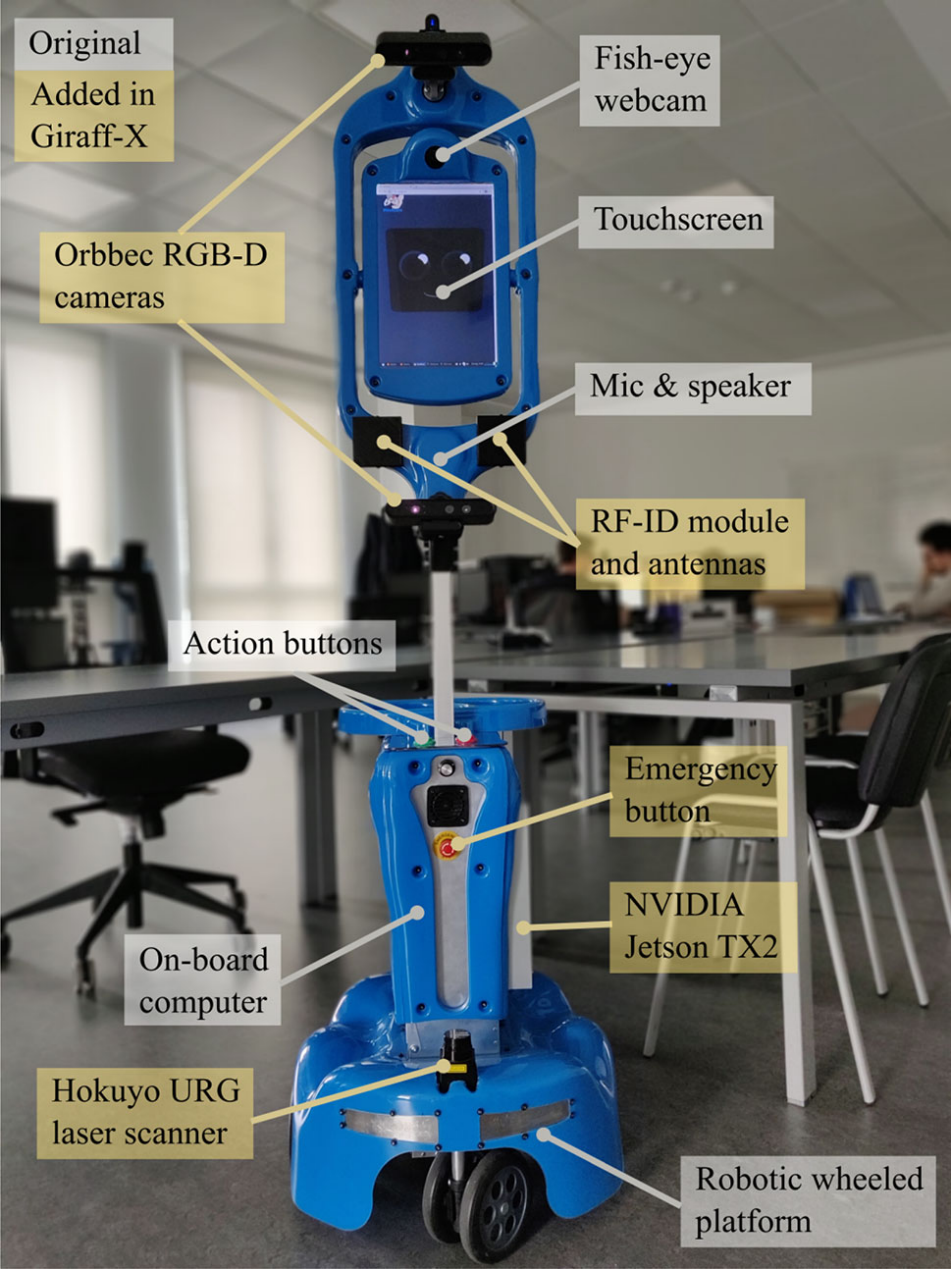
The Giraff-X mobile robot

The robot, called Giraff-X, is the main actor of the system, embodying the virtual caregiver (Sect. 4.5) at the elder’s house. It is an enhanced version of the Giraff teleoperated robot developed explicitly for AAL [9, 11, 47]; it has a height comparable to humans (1.70 m), a display on the top, and a two-wheel differential drive with two caster wheels that allows it to turn in place (see Fig. 3).

Giraff-X perception capabilities are based on two RGB-D Orbbec cameras (Orbecc Astra) on top and bottom of the screen frame, a 2D lidar (Hokuyo URG) placed on the robot base facing the front, two RFID receivers located beneath the screen and a fish-eye camera attached to the top. Finally, as some tasks may be computationally intense (e.g., relocalization and person identification), a GPU board (nVidia Jetson TX2) has been added inside the robot base to leave the CPU (Intel i7-3610QM) free to manage the main robot’s functionalities, thus implementing a hierarchical control.

For further details on the robot’s configuration and its provided services please refer to [40].

**Fig. 4:**
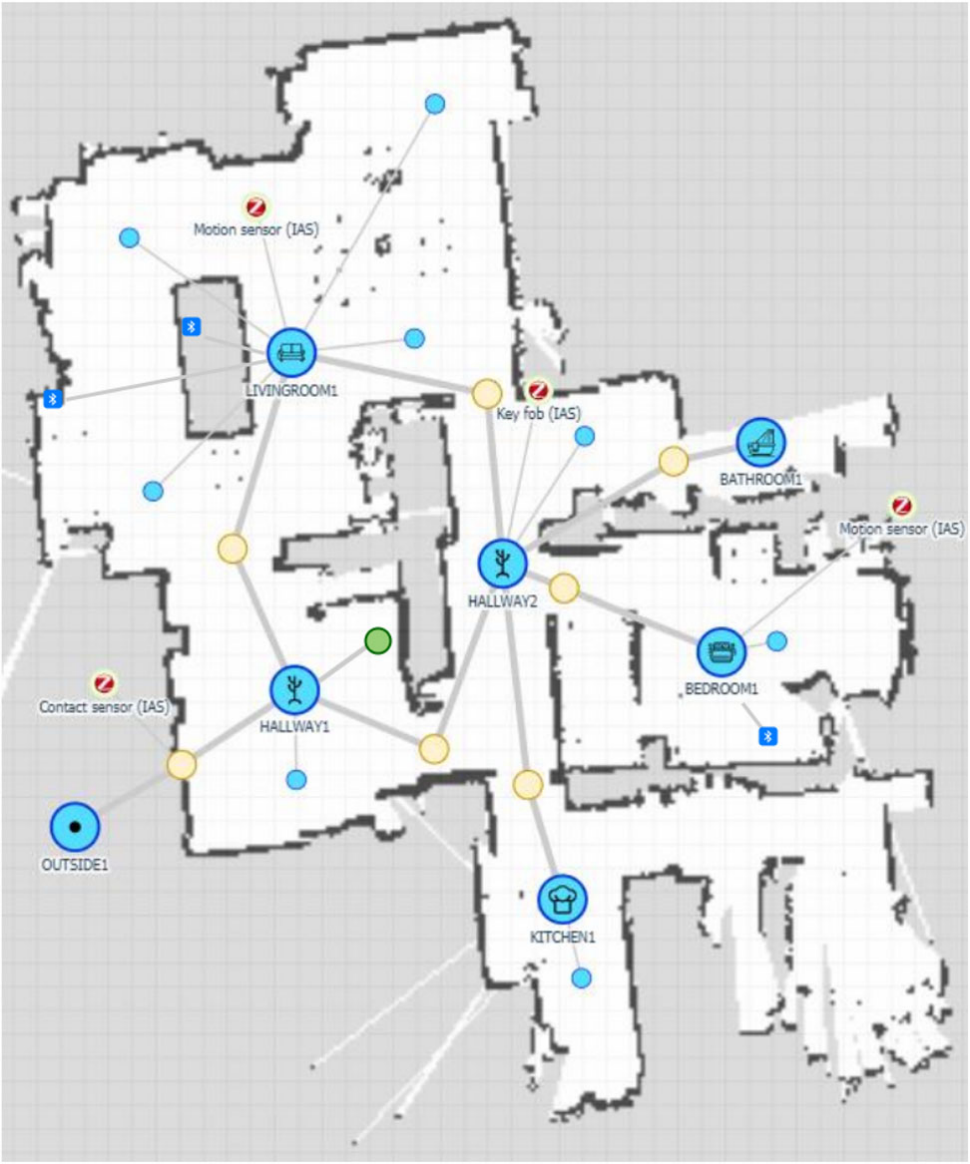
Example of an annotated map of the working environment. Blue circles with a logo represent topological places such as rooms and hallways, yellow circles correspond to doorways, and the smaller light-blue circles are topological locations inside the rooms that the robot can reach during navigation. The robot’s charging station positions is marked in green, while red and dark-blue marks indicate the position of the environmental sensors

#### Autonomous Navigation

The control stack is based on the Robotic Operating System (ROS) that provides the middleware for supporting the whole software architecture. Widely-used standard ROS nodes (e.g., MoveBase, AMCL, etc.) and specifically implemented and third-party ones (e.g., OpenNI, PCL, or OpenPose) have been integrated into the ROS robot framework.

A 2D map of the apartment is built at installation time [25, 40] from the lidar data. Such a map provides the basis for navigation, and it is enriched with the location of key points (e.g., the position of the docking station and of the environmental sensors, see Fig. 4).

The robot moves from one key point to another using the navigation functionalities offered by the ROS navigation stack^5^. Navigation is made more robust to obstacles by sampling clouds of 3D points over the objects in front of the robot and projecting them onto the floor plane. This 3D point cloud allows performing obstacle avoidance that accounts for the unexpected cases when the 2D lidar localisation fails or when the robot is manually moved, e.g., by the user or by a caregiver [22], making navigation reliable and safe.

Doorways are critical in real apartments as they may offer narrow passages. This is taken into account with an *ad hoc* method based on an automatically annotated map [45] whose example is depicted in Fig. 4. When the robot is required to navigate to the user, it first searches in the last room they were detected through Passive InfraRed (PIR) sensors (Sect. 4.2) using the method presented in Sect. 4.5.4. A navigation path is then computed so that the robot can approach the elder in an ecological way (complying with proxemic constraints), as described in [22].

#### Human Robot Interface

Particular care has been devoted to developing the interfaces of the robot, to achieve a smooth interaction with the user. Speech dialogues have been chosen as the primary interaction modality since they are considered the preferred mean by older adults and the most natural modality in general [40]. To this aim, conversations are driven by the robot’s Dialogue Manager (DM). Following a finite-state-machine approach, this module keeps track of the current state of the interaction by representing it as a selection of domain-specific keywords selected from a pre-defined vocabulary. State transitions are dictated by the user utterances (and the messages extracted from them) that the DM receives.

Speech recognition is implemented in two steps: the user utterances are picked up by the robot microphones in real-time. They are then translated into text through Google Cloud Speech APIs with a delay estimated (from trials) to be less than 100 ms. The DM identifies keywords related to each dialogue instance according to the state it is in and processed inside the semantic domain of the current particular interaction. Feedback is produced with Acapela Voice as a service^6^ that allows having a human-like speech output, a fundamental feature for a positively perceived interaction.

Given the robustness of the technologies employed for voice recognition and synthesis, the resulting performance was good in most cases. However, some issues were detected in certain apartments related to a poor internet connection, as the robot relies on external Speech-To-Text cloud services. To cope with that, we adopted the following procedure: the robot asks a question to the user and if, after a timeout (30s), no answer is received from the cloud, the question is repeated. If no answer is received after a second timeout (30s), the robot apologises to the user (by saying “Sorry, I can not understand”) and the VC reschedules the intervention as it could be a temporal connectivity issue. At the same time, the user could answer using the two green/red buttons placed on the robot to give a positive/negative answer. The use of buttons bypasses speech recognition in those cases.

In addition to the speech interface, a visual interface is displayed on the robot’s screen, whose design has been refined to improve the user’s experience according to feedback collected from preliminary tests with end-users. In its final configuration, the robot displays on the screen a pair of blue eyes on a dark background. In order to give an impression of “humanness”, the robotic eyes are characterized by micro-movements. The robot’s display is also used to show subtitles whenever the robot speaks, to facilitate understanding.

#### Robot Functionalities

The Giraff-X robot provides a set of services as the main intelligent actuator of the system. During normal operation, Giraff-X waits at the docking station until the system triggers an *intervention* prescribed by a suitable *scenario*; then, the robot, under the supervision of the VC, autonomously performs the adequate temporal sequence of actions:

1. undocks (if necessary),
2. safely navigates to the expected user location [45] (updated by the system in real-time, see Sect. 4.5.4),
3. locates the user and, if not found, performs a search in the apartment,
4. finds the user within the house, and approaches them taking into consideration the proxemic constraints [22],
5. interacts with the user to carry out the specified action,
6. provides feedback to the VC,
7. if there is no other intervention planned within a short time, it returns to the docking station.

The robot’s primary purpose is to perform this procedure autonomously with the only supervision of the VC (i.e., without any help from the elder or an operator) by navigating in the environment. Given this, the fact that the robot is perceived as safe and unobtrusive by users is crucial. The robot can perform three different types of services: tasks requested by the system, tasks requested by the user, and self-management tasks.

**Robot tasks requested by the system**. These services are triggered automatically by the VC according to a schedule decided by a real caregiver (Table 1) and are: *reminders*, where the user is asked to perform a task (e.g., measure their weight), or *invitations*, which inform the user about the possibility to perform an activity such as playing a social game on the CBAC (Sect. 4.4). These functionalities require the robot to look for the user within the house and interact with them.

Moreover, the robot is used to gather monitoring data produced by interactions with the user. They are (i) *spot questions*, vocal interactions where the robot asks something and listen to the user’s answer to it; (ii) digital versions of paper-and-pencil cognitive tests, commonly used for neuropsychological assessment, Bells, TMT-A, and TMT-B, that are administered by the robot through the tablet, following an approach improved from that of [42] [36], and [41].

**Robot tasks requested by the user**. These services are activated upon user request and are (i) the *call for help* and (ii) the *search for lost objects*.

When a user calls for help from anywhere inside the house, the help request recognised by the closest microphone (Sect. 4.2) and sent to the VC (Sect. 4.5). This activates the robot that starts navigating the environment in search of the user. When the user is identified, the robot asks to confirm the call for help. If the emergency is confirmed or no answer is received, the system establishes a communication with the caregiver, who can activate a video call or take remote control of the robot to actively assess the situation inside the house by teleoperating it and checking its camera feed.

In the *search for lost objects* service, the user commands the robot to search a specified object to which an RFID tag has been previously attached. Giraff-X exploits either its RFID antennas (computing a rough estimation of the object’s location) or computer vision (to recognise the object obtain a more accurate localisation of it [64]). The service is requested using a web application embedded into the CBAC component (Sect. 4.4) from which the user can select the RFID-tagged object to search for.

Finally, the user can interact with the robot through the microphones distributed within the apartment by using two vocal commands: *“go home”* cancels robot’s current intervention and triggers an autonomous docking, and *“come here”* who calls the robot to reach the user.

**Self-management robot tasks**. It includes a set of functionalities to ensure a proper autonomy level of the robot, such as managing the battery level, detecting idle periods while not charging, shutting down the screen when not in use to save energy, or performing an autodocking action if it has been idle for a long time or its battery level is below a critical threshold.

**Robot Recovery Behaviour**. During the execution of its daily tasks, the robot may encounter some issues. If the robot cannot complete its task autonomously, it informs the VC about the possible cause of the problem and automatically starts a self-management task to return to the docking station. If the problem prevents the robot from reaching the docking station, and after three failed docking attempts, the robot asks for help (by voice) to the user. The user is then instructed by Giraff-X to either (i) assist with the problem (e.g., removing objects interfering with the robot navigation, like a chair or a closed door), (ii) to manually move the robot towards its docking station, for which the robot integrates a red mushroom head push-button (see Fig. 3) to disable the robot’s motors, or (iii) to contact a technician. See [43] for further details.

**Fig. 5:**
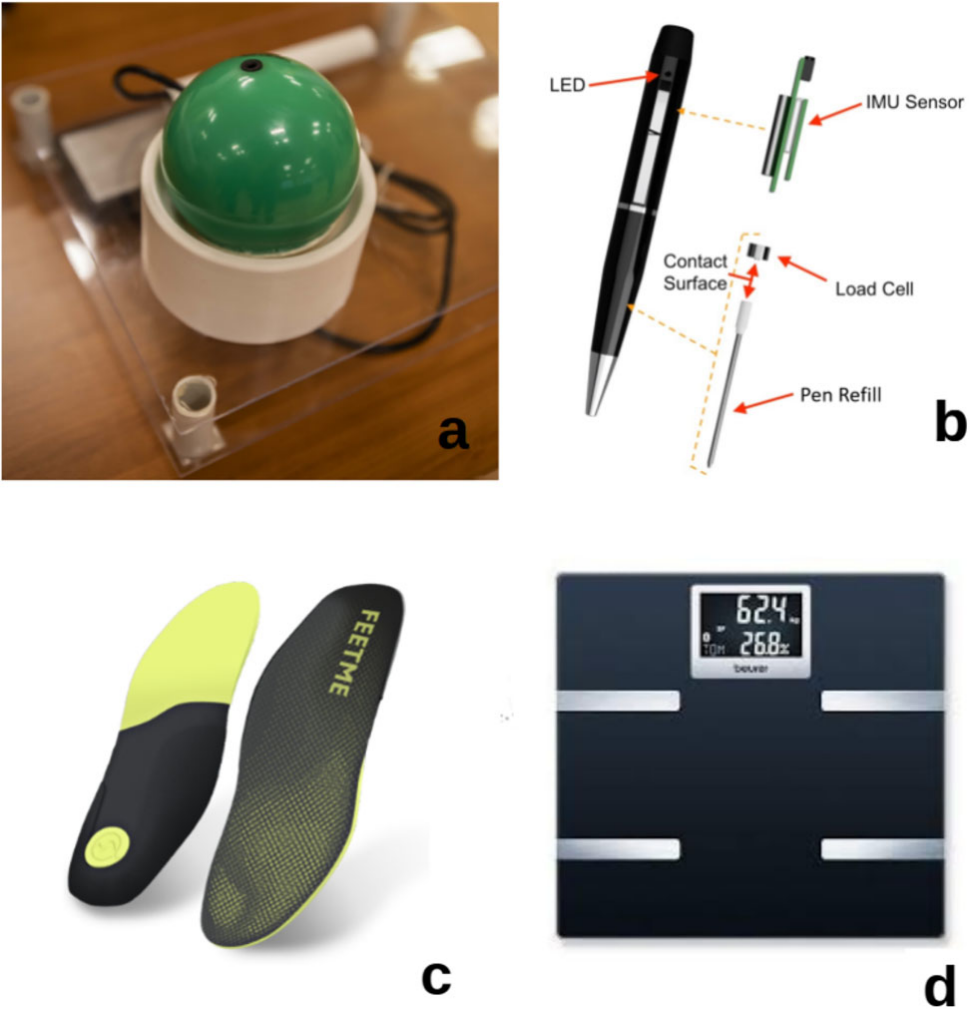
The smart objects of MoveCare: (a) the anti-stress ball, (b) the ink pen, (c) the insoles, and (d) the Bluetooth scale

### Environmental Sensors

Environmental and user data is collected from a set of domotic sensors integrated inside an Internet of Things (IoT) network, including thin accelerometers to identify when the elder is sitting on the sofa or lying in bed, a smart plug to detect when the TV is on, a switch sensor attached to the main entrance door of the user’s apartment to detect when the user is opening it, PIR sensors to detect presence inside specific rooms, and *microphones* to pick up in a robust way the call for help request and the user’s vocal commands aimed at the mobile robot.

The microphones are designed to adapt themselves to the noise level and elder voice pitch and loudness, implementing a Zero Crossing Rate and short-term Energy methodology based on [49]. To this aim, an advanced Automatic Speech Recognition (ASR) firmware processes in real-time the voice picked up by the microphone, recognising the set of predefined commands through a discrete symbol Hidden Markov Model [21]. Microphones are spread through the apartment to detect user requests from any location. Therefore, the necessary number of microphones is correlated with the apartment dimension and structure. On average two to three microphones have been required.

In addition, the IoT network integrates a Master Switch that allows users to turn off the whole system. This functionality aimed to be used when guests are present, both for privacy reasons (guests probably did not agree to participate in the study) and technical reasons (data analysis has been designed to consider only one person in the environment). When the system is turned off, the robot is deactivated, and no data is collected.

All sensors communicate wirelessly to a *concentrator* that provides a gateway to the cloud. The protocol adopted is MQTT^7^. The gateway can make automated phone calls to deal with a call for help, even when a transient Internet connectivity failure occurs. The concentrator also stores the map of the environment (as acquired by the robot during installation) and the system’s setup.

### Smart Objects

The MoveCare platform enriches the user’s data collected by the environmental sensors through a set of sensorised daily-use objects referred to as *smart objects*. They enable collecting measurements relatable to physical and cognitive decline and frailty (that are difficult to collect otherwise) or provide specific assistive functionalities. All these objects have in common that the user needs to actively use them in order to acquire measurements, as opposed to the environmental sensors described in Sect. 4.2.

Four objects of everyday use have been integrated inside the platform (Fig. 5): a *smart ball*, an anti-stress rubber ball to measure the maximum grip force [34], an *ink pen* that allows to normally write on any sheet of paper while collecting data on tremor and degeneration of handwriting [35], a *bluetooth scale*, with which recording the daily weight of the user, and a pair of commercial *sensorised insoles*^8^ to evaluate gait degeneration. These objects are designed so that the interaction of the user with them is natural, and can provide at the same time valuable monitoring data in an unobtrusive and ecological way.

One of the desirable features of smart objects is long battery autonomy. To this aim, we have limited the use of radio transmissions: the ball and ink pen’s on-board processors are programmed to be woken up and start their data acquisition/transmission cycle when an object’s movement is detected. These objects upload data to a host; when the host is not within range, the data are retained on-board for later transmission.

The insoles were meant to be used with the user’s smartphone as data-receiving host, where a specific app had been installed. This app has been designed to activate gait recording in two different modalities: manually, for the expert user, or automatically based on GPS. In the latter modality, when a predefined distance from the user’s home is reached and detected through GPS, the app automatically connects to the insoles and starts recording gait data. Likewise, when the user returns towards home, or when a preset maximal number of daily steps is reached, the recording is stopped. Gait data is then stored on the cloud, and automatic post-processing obtains daily spatio-temporal parameters of gait.

### Community-Based Activity Centre

The Community-Based Activity Centre (CBAC) [39] has been designed to push elders to perform, together with peers, cognitive and physical activities with the aim of promoting an active lifestyle and fostering socialisation. Its functionalities are motivated by the fact that loneliness and social exclusion are critical factors for frail or pre-frail older adults. CBAC is a modular web application composed of activities coordinated by a supervisor providing access to them. The server-side has been developed in NodeJS, a framework based on JavaScript, the client views are based on HTML5 and JavaScript, and the app exploits the WebSocket protocol to provide real-time responsiveness.

The client-side can be run from a web browser and, in MoveCare, it was deployed in each user’s apartment on a tablet and a TV set-top box connected to a TV screen and browsed through a remote TV controller. Both tablet and TV set-top box were provided to the user.

**Fig. 6:**
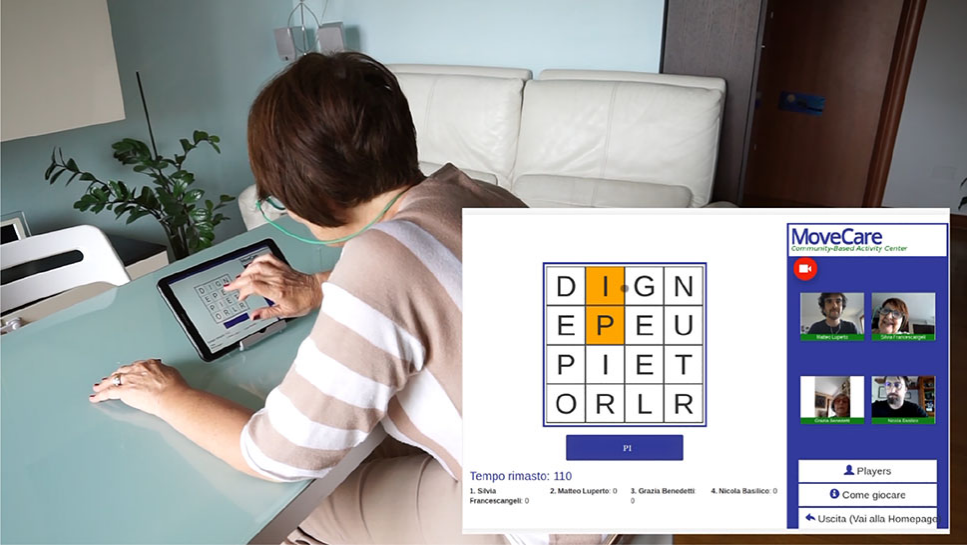
A participant to the pilot playing with the CBAC on the tablet setup

On the tablet, CBAC offers a set of cognitive games to the users: two cards games (scopa and briscola, popular in the South of Europe), a word game, a drawing game, and a puzzle game. Physical activities are aimed to keep elders in good physical condition. To this aim, we have set up four digital video channels that show a real teacher who guides the elder through gentle exercises. Clinicians have designed the exercises to improve balance, muscle strength, and elasticity, to decrease the risk of falls. All these activities are carried out in group sessions taking place in virtual rooms. The room layout, rendered in the client view, shows on the left side a virtual deck on which activities are carried out and where events (e.g., game moves, drawing marks) are synchronised in real-time between participants. On the right side, the live audio-video of the participants is displayed. This stream is transmitted peer-to-peer through a WebRTC channel (Fig. 6).

A more challenging set of physical activities is offered through a set of exergames designed and developed for postural rehabilitation and tested in that domain [52]. Such exergames are accessible through the TV setup and are played with a Wii balance board. By shifting their centre of balance while standing on the board, users induce a change in its pressure. Pressure changes are mapped to the control of an avatar displayed on the screen and used to accomplish the exergame’s goals.

Besides the two main groups of activities described above, CBAC also integrates:

- a catalogue of geo-localised outdoor events to which users can subscribe individually or in groups; this tool is aimed at stimulating elders to go outdoor, possibly with peers;
- a set of screening neuropsychological tests developed as digital versions of the well-known Bells, TMT-A, and TMT-B cognitive tests. In the Neuropsychological Tests scenario, they are administered through the tablet under the supervision of the robot as explained in [38];
- an interface to start the Finding Lost Objects scenario, from where the user can select the object to be searched by the robot;
- an interface to teleoperate the robot in the Call for Help scenario.

The execution of cognitive and physical activities in CBAC produces activity reports that summarise usage statistics (frequency and time of usage, involved peers) and activity-dependent performance indicators (scores). This data is stored in a cloud repository and accessible for analysis to the VC through a set of APIs.

### Intelligent Virtual Caregiver

The Virtual Caregiver’s (VC) role is to analyse the data provided by the environmental sensors, the smart objects, the CBAC, and the service robot to assist and encourage the usage of the adequate components of MoveCare. To this aim, it computes suggestions about possible activities and, when applicable, peers to play with. It also computes trend indicators and provides the real caregivers with regular reports and warnings.

The VC coordinates the heterogeneous MoveCare system in the execution of the MoveCare scenarios and actively triggers their execution through interventions.

**Fig. 7:**
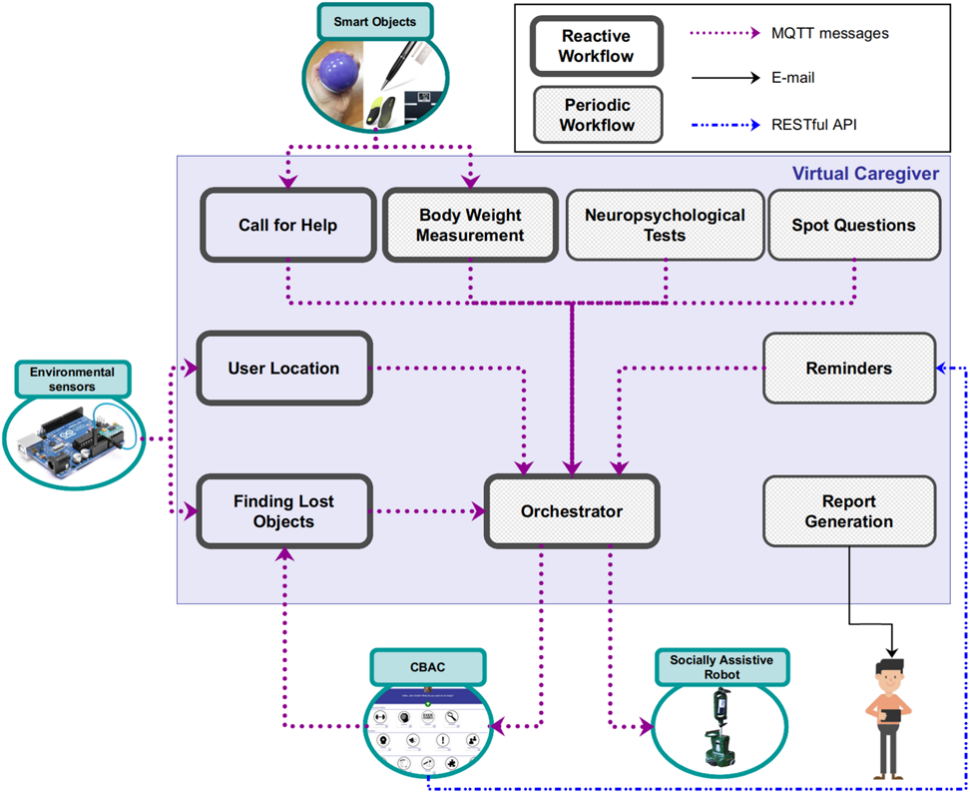
The architecture of the Virtual Caregiver. All the modules implementing the periodic workflows query data from the system’s central database. This connection has been omitted from the figure for the sake of readability

The structure of the VC is presented in Fig. 7. It has been designed around the scenarios presented in Table 1, and it contains 9 modules associated with the scenarios in which it is involved: “Call for help”, “Body Weight Measurement”, “Neuropsychological Tests”, “Spot Question”, and “Finding Lost Objects” modules implement the logic of their respective scenario. The “Reminder” modules aggregates the functionalities of the “Grip Force Measurement”, “Social Cognitive Games”, “Gentle Exercises”, “Balance Exergames”, “Outdoor Suggestions”, and “Use of Smart Objects” scenarios. Indeed, for these scenarios, the VC’s involvement is to send reminders to the user when the scenario has not been played for a given amount of time. In addition to these scenarios modules, the VC includes 3 utility modules: “Report Generation” for compiling and sending reports with indicators to the real caregivers, “User Location” to keep track of the current room where the user is located, from environmental sensor readings, and “Orchestrator” a model that manages the interventions generation with the objective of not causing excessive disturbance to the user (limiting interventions and reminders frequency during the day, and suspending them during user’s rest time).

The modules in the VC may follow two not mutually exclusive workflows: the *Reactive Workflow* and the *Periodic Workflow*. The reactive workflow is activated each time the module receives data from another component (obtained by a publish-subscribe communication infrastructure based on the MQTT protocol). This data is then immediately analysed, and the appropriate intervention might be generated. With the periodic workflow, the module is woken up at pre-determined times (usually defined with some regular frequency, e.g., daily or weekly), during which it collects from the database the new data generated since its last activation. Such data are then analysed to evaluate the generation of required interventions.

In the remainder of this section, we will provide some details about VC’s modules and their role in the various scenarios. A preliminary version of this component has been presented in [55].

#### Scenarios

The Call for Help module is purely reactive , awaiting the microphones to pick up a vocal request for help by the user. When such a trigger is activated, the module initiates an escalation process: first, an intervention is generated to ask the user to confirm the help need. The robot performs this step and, upon reaching the user, asks him to confirm the request. If the user does not confirm, then the request is archived as a false positive, and the scenario is aborted. If the user does confirm or does not answer, the escalation proceeds and a phone call with an automated alert message is placed to the user’s caregiver. Through a dedicated section of the CBAC, the caregiver can then control the robot to manage the emergency. With such control, the caregiver can move the robot around the apartment and, at the same time, establish contact with the user through a video call hosted on the robot itself. The Call for Help scenario is then logged as a true positive and, the VC will later include it in the periodic report for caregivers.

The Body Weight Measurement module implements both the periodic and reactive workflows. The periodic workflow is activated weekly and checks if the user measured their body weight at least once during the past week. If not, the module generates a reminder for it. In the reactive workflow, the VC analyses the data gathered from the smart scale to detect anomalous weight variations (more than 2% of the previously registered weight). If such a change is detected, the VC generates an intervention to ask the user to repeat the measurement. If the change is confirmed, an alert is included to the caregiver in the periodic report.

The user is expected to regularly perform two neuropsychological tests commonly used to detect early signs of cognitive impairment [36]. The Neuropsychological Tests module implements the periodic workflow exclusively and is activated daily. Its role is to supervise the administration of cognitive tests. Moreover, it checks when the test was last performed, and if the date for a new test has arrived, it triggers an intervention for the user to perform the tests after a given amount of weeks (this amount is a requirement identified by the clinicians).

The Spot Question module implements the periodic workflow and is activated daily. Its role is to select one or more questions from a pool, according to a pre-defined frequency decided by clinicians. The questions can be related to the user’s recent activities, the current context (day or month), or an event in the user’s past. Spot questions are aimed at assessing episodic and prospective memory as well as spatial and temporal orientation. If the question relates to the user’s recent activity (e.g. “Can you tell me if you played cards in the last 3 days?”), the Spot Question module queries the MoveCare database to retrieve the correct answer from historical activity data.

This Finding Lost Objects implements the reactive workflow. It is activated when the user triggers the associated scenario from a dedicated CBAC section. Upon receiving the user’s request for searching a selected object, the module generates an intervention to start the task execution by the robot.

#### Reminders

To promote the use of the platform, the system provides reminders to the user to perform a recommended scenario if it had not been done for more than a pre-defined amount of time. (This requirement about the minimum frequency of usage is determined as a requirement by the clinicians.)

The VC’s Reminders module implements the periodic workflow and is activated daily. It applies to the “Grip Force Measurement”, “Social Cognitive Games”, “Gentle Exercises”, “Balance Exergames”, “Outdoor Suggestions”, and “Use of Smart Objects” scenarios. Each one is associated with a priority representing how important the scenario is for the user’s well-being. If several scenarios simultaneously need a reminder, those with higher priority are given precedence (ties are broken randomly).

In addition to reminders, this module also provides “positive feedback” when scenarios are performed regularly and by following the indications.

#### Report Generation

The Report Generation module implements a periodic workflow and is activated weekly. Its role is to inform the caregivers responsible for each user of the week’s most meaningful events, namely abnormal weight changes and the occurrence of calls for help. In addition, the report contains the answer given to the Spot Questions asked during the week, which a clinician to whom the report is handed could analyse to follow the evolution of the users’ state. Upon activation, the module queries the relevant data from the database and sends it by email to the clinician responsible for the user. This module also considers the past data related to the scenarios with a large time interval view of the event. For instance, while the Body Weight Measurement module analyses each measurement with regards only to the previous one, the Report Generation module considers all recorded measurements over several weeks to detect possible critical variations. To assess if weight variation is critical, its rolling average is computed over a window of 14 days, and a straight line was fitted to the data. If its slope is higher than a given threshold, then the alert is raised in the report.

#### User Location

The User Location module implements the reactive workflow. The role of this utility module is to track the room in which the user currently is so that the robot can navigate efficiently. To do so, it gathers information from the PIR sensors (Sect. 4.2) and each time a PIR sensor changes its on/off state, the VC updates the user’s location. This is done by combining the data from the PIR and the door sensor with the a priori knowledge of the apartment map where the position of the installed sensors are semantically annotated (see Fig. 4). If the user’s presence cannot be perceived anywhere in the house (all the sensors are off), their location is marked as *outdoor* if the door sensor has been activated recently. It is set to *unknown* otherwise. This might happen, for example, when the user is in a room that is not monitored (e.g., the bathroom) or too still for the sensors to be activated (e.g., sleeping).

#### Orchestrator

It is essential to ensure that the interventions generated by the system do not overload the user and that they are delivered at an appropriate time (e.g., not while the user is sleeping) to improve user-friendliness and acceptability. Timing the interventions and monitoring their delivery is the role of the Orchestrator module, which leverages a set of rules approved by the clinical partners to improve the user’s experience. These rules act as constraints that the clinicians can add or depend on the user’s detected activity.

The Orchestrator module implements both the reactive and the periodic workflows. It is called each time the VC module sends an intervention request and every hour between 08:00 and 21:00 in order to process potentially queued interventions. Due to the periodic nature of the VC module, the list of pending interventions is cleared every evening and new interventions are re-generated by the module on the next day, if needed. The Orchestrator operates based on priorities that ensure that the most critical interventions are sent first, and contextual rules and predefined constraints (summarised in Table 2) to ensure that the user is not disturbed by too many interventions too often. In the reactive workflow, the Orchestrator is activated each time an intervention is received. The intervention is stored in a list of pending interventions, sorted by priority. The Orchestrator then checks if all the rules and constraints are satisfied, in which case it sends out the intervention with the highest priority. In the periodic workflow, the Orchestrator is activated every two hours, performs the check of the rules and constraints, and send the intervention. It is important to note that two types of interventions are exempt from checking the rules and priorities: interventions related to the call for help and the finding lost objects scenarios. These interventions are indeed sent following a request from the user. Therefore they are processed immediately and sent regardless of rules, constraints, and priorities.

**Table 2:**
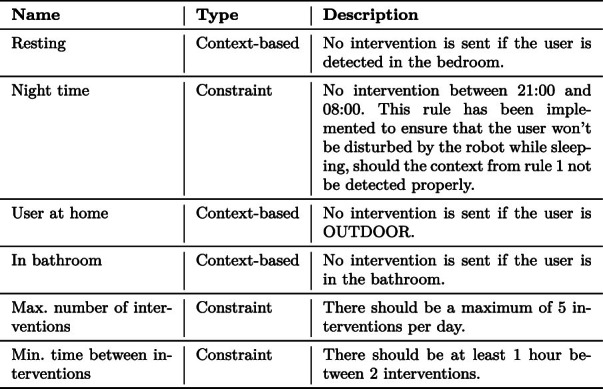
Rules and Constraints implemented in the Orchestrator

## Results

This section evaluates our proposed framework’s long-term feasibility and acceptability by presenting the experimental results obtained from the pilot test, where the system was installed in the own apartment of elders living alone and used for 10 weeks each. The pilot was organised as a prospective, multi-centre, feasibility study carried out in Spain and Italy.

Not all the elders received the entire platform; some received the system with the robot (with-robot) while the others received the platform without it (without-robot). This allowed us to test the impact of the presence of Giraff-X (the “face” of the Virtual Caregiver) on acceptability and feasibility.

**Fig. 8:**
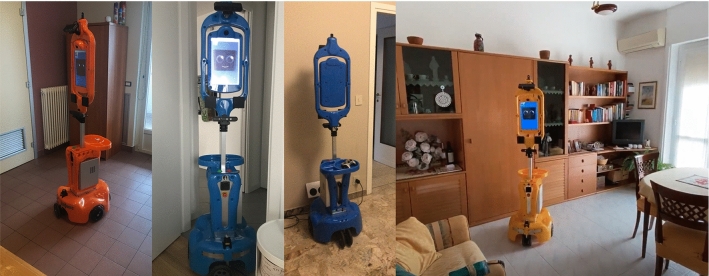
Giraff-X carrying out various tasks inside the apartment of the pilot experimental campaign

### Recruitment

A total of 25 elders have participated, 11 of them have been recruited in the area of Milan (Italy) while the remaining 14 in the city of Badajoz (Extremadura, Spain). The participants from Milan have been divided into two groups: 7 people were living in their own apartment (we shall denote them as group ITA-HOME), while the remaining 4 were residents of independent apartments hosted by KORIAN^9^, an Assisted Living facility in the Province of Milan (we shall denote this group as ITA-AL). All the participants from Badajoz formed a single group and lived in their own apartment (we shall denote this group as ES-HOME).

The recruited participants were outside the frailty state and living in apartments where the system could be deployed without significant interventions. These conditions were captured by the application of the following selection criteria:

- $$\ge $$ 65 years old;
- living alone, without pets, in a house without stairs and rugs, receiving daily assistance for no more than 1 hour per day;
- Mini-mental State Examination (MMSE^10^) score $\ge$ 26;
- $\le$ 2 points in Fried criteria [10, 20] or robust people: 0 points in Fried criteria but with GDS (Geriatric Depression Scale) $\ge$ 9 or UCLA loneliness scale > 35 [58];
- keen to use technology;
- high-speed Internet connection available at home.

Moreover, sensory deficits (deafness, blindness) or motor disability (paraplegia) potentially precluding using the system were considered exclusion criteria.

The recruitment campaign started one year before the pilot. In Spain, where people lived in a low-populated area, an extensive advertisement campaign was conducted through media and meetings to maximise outreach. People who showed interest were included in a candidate list, and they were later called for a final selection interview. In Milan, where people lived in a highly-populated area, recruitment was carried out through a regional association of volunteers, ANTEAS^11^, and among the patients of the Geriatric Unit of the Policlinico of Milan hospital^12^.

The profile of the recruited users is the following. The average age was 76.7 years ($\sigma =7.2$; $median= 78.5$, Range: 65-92;). The participants in Italy were slightly older (average age 79.1 years) than the Spanish participants (average age 75.9 years). This is because the ITA-AL facility users were older than the other participants (average age 84.8 years). Moreover, their MMSE average score was lower (average of 27/30) than that of other elders, 28.75/30. ITA-HOME participants age (average age 74.7 y) was similar to the one of ES-HOME.

### Pilot Organisation

The pilot was organised in two rounds of 15 elders each. The first round, R1, was carried out from September 2019 to December 2019. The second round, R2, was from January 2020 to March 2020. After the first round, 5 elders from R1 asked to continue the experimentation in R2, and therefore used the system for both rounds, R1+R2 as can be seen in Fig. 8.

A total of 13 elders used the whole system, including the robot (they were initially 14, but one of them decided to drop out from the study). We shall denote this condition as with-robot. On the other side, 11 elders received the platform without Giraff-X, a condition we call without-robot. Such a differentiation originated from practical contingencies of the pilot: some users agreed to participate only without having the robot deployed at home; some others instead could not receive it due to a limited number of available platforms. We took advantage of these two different setups to obtain a between-subjects comparison of the two conditions, from which to assess the impact of the robot’s role in the same system.

A complete overview of how participation unfolded with respect to groups, rounds, and robot condition is detailed in Table 3.

**Table 3:**
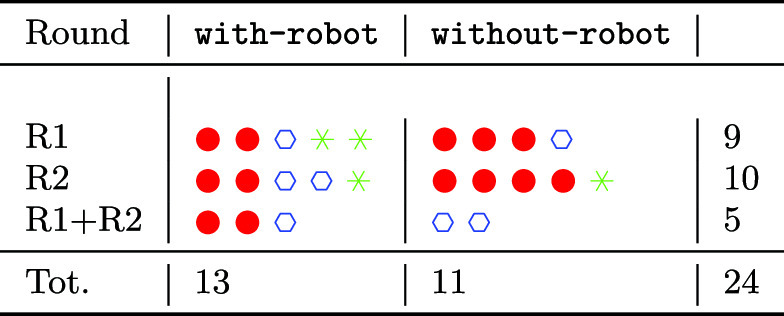
List of users divided by group and by pilot round.  are users from ES-HOME,  are users from ITA-HOME, while  are users from ITA-AL

To provide support in the preparation and during the two rounds, a support team constituted of a clinician and a technician was formed in each of the pilot sites (Italy and Spain), with the goal of providing functional and technical support, respectively.

#### Functional Support

The clinical members of the support teams introduced the platform to the elder after its installation, providing a detailed explanation of the system functionalities, how to use them, and how to approach possible malfunctions. For example, users were instructed how to answer the requests of assistance from the robot in case it was not able to fulfil a task (e.g., due to a blocked path) by following the indications provided by the robot itself [43] (move the robot to the docking station or call a technician). During the pilot, the clinical members were also the primary referents for any issue as the technical partners trained them on the platform to best illustrate its operation to the elders.

Moreover, user manuals for the components and the whole system were provided: they have been designed with simplicity in mind and primarily contained visual material to make their consultation quick and intuitive.

#### Technical Support

The technicians responsible for the installation were in charge of identifying any technical issue in the pilot and try to solve it. If the issue could not be solved, it was forwarded to the University of Milan’s research unit, where the system assemblage and testing had taken place. The unit coordinated this second line of technical assistance, eventually connecting with other units in charge of specific components.

Topic-based instant messaging channels were exploited to efficiently coordinate the research units in dealing with technical issues and provide responsiveness, where the first line of assistance could provide requests to the second line. A centralised log of major issues was maintained to allow, from one side, to shorten the intervention time when the same situation occurred again and, from the other, to debug the system and release updates. A graphical tool for remotely monitoring the different installations was developed to show (in real-time or in replay mode) the time series of events the system went through during the execution of any scenario [43]. This information, useful identify communication problems or of transient failures, is particularly critical for the maintenance of heterogeneous and interconnected systems like ours, where problems signalled by the users might result from complex and diverse chains of events.

We have also adopted tools to access the system remotely, particularly custom remote access to the IoT concentrator. These tools turned out useful to assess the network communication and sensors’ status and to provide updates and restarts. A remote desktop application was installed on the CBAC TV set-top box and on the robot to grant access in case of needed updates, bug fixes, or system restarts.

#### Deployment Modalities

A first setup phase was carried out inside the lab to configure the network access of the devices, install the software, and create the user accounts.

In the second phase, the refinement of the system was carried out on-site by a team of one computer scientist and one clinician. This phase lasted approximately half a day on each installation. First, the robot was teleoperated through the house to create a map of the environment (Fig. 4b), which was then automatically uploaded to the concentrator. The map was then semantically annotated with the optimal positions of the robot inside each room (large blue circles in Fig. 4) and the installation locations of the environmental sensors (at least one PIR sensor was placed in each room).

During this setup phase, the clinical member of the installation team trained the elder to use the system. Due to the presence of several components, training was divided into two sessions, lasting one to two hours each, for each user. If needed, the users could request further training sessions. Robot functionalities were demonstrated to users by performing two scenarios with technical supervision (a spot question and a weight measurement) and answering the users’ questions about the robot behaviour.

After R1, issues that arose were analysed. Many of them required minor fixes in the apartment (e.g., better placement of sensors or fixing pre-existing TV settings for better compatibility with CBAC). More critical issues involved network availability, whose shortfalls often caused timeout and slowed-down operations. These issues were solved with a revised version of the software that checked periodically for network connectivity to warn the user upon losses. Moreover, minor functional modifications to respond to unexpected events that happened during R1 were introduced. For example, we removed the possibility for the robot to move at night unless an emergency was triggered. This was due to unexpected circumstances that provoked the robot to move at night, trying to perform a docking manoeuvre after being disconnected from the docking station by an unexpected event (as a bump). Microphones were also updated to reduce false positives in commands due to external noise that, despite being limited in number, were signalled by users. These improvements introduced between the two pilot rounds allowed, as expected, getting a more robust system with a better appreciation by the users.

All data was anonymised to ensure users’ privacy and data access was available only to authorised users. Voice and video data (e.g., commands received by microphones or images of the users collected by the robot) were never stored, being processed locally and directly on the media in charge of their collection. During an emergency scenario where a caregiver remotely controlled the robot, video-chat communication in the CBAC was not stored. Users can ask to inspect data collected by the system and, upon request, delete them. Moreover, users were provided with a master switch button (see Sect. 4.2) to turn off the system, including monitoring functionalities, whenever they desired so. Users also signed an informed consent regarding data protection and management, as indicated in the Ethical Committee approval of the pilot study.

**Table 4:**
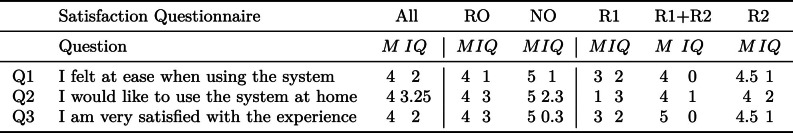
Satisfaction questionnaire. *M* is the median, *IQ* is the interquartile range, RO is with-robot and NO is without-robot

**Table 5:**
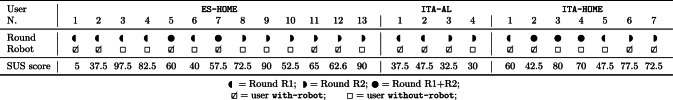
Overview of the pilot condition for each user and results of the SUS questionnaire. (Customarily SUS is considered as positively evaluated when the score is above 68)

### Evaluation Methodology

Pilot users evaluated their experience with the platform through a set of specific questionnaires. The evaluation was performed using a set of Likert scales, where each Likert item was a question with answers in a 1-to-5 numerical range, using 1 for “strongly disagree” and 5 for “strongly agree”.

Questionnaires were related both to the whole platform and the evaluation of specific components; users were also allowed to make open comments if they desired so. Answers to open-ended questions were used to better explain this user’s evaluation of the different items. We provide here results obtained in questionnaires related to evaluating the platform as a whole, as those allow us to evaluate the long-term impact of Giraff-X. (Note that we omit the questionnaires evaluating single specific components and scenarios as those are not of interest for the evaluation provided here and are presented elsewhere [39].)

The following questionnaires were used (specific questions are given in the appendix):

- a satisfaction questionnaire constituted of only three questions (1. I felt at ease when using the system; 2. I would like to use this system at home; 3. I am very satisfied with the experience.). Three open-ended questions were also provided (Sect. 5.4.1 and Table 6, in the Appendix).
- A usability questionnaire. To this aim, the widely used SUS [6] has been adopted (Sect. 5.4.2 and Table 7, in the Appendix).
- A system validation questionnaire made up of 16 questions (Sect. 5.4.3 and Table 8, in the Appendix).

We used a non-parametric U Mann Whitney test to compare the results in different conditions (with-robot/without-robot) and a Pearson’s Chi-squared test to assess any association between the answers to different questions. The comparison of with-robot/without-robot condition was between-subjects. The issues related to organisation, logistics, support, and deployment outlined in the previous sections represent built-in overheads for this kind of experimental campaign. They should never be neglected or underestimated, especially when dealing with a long-term perspective. For this reason, the number of participants we involved (our sample size) could not be larger than the one we are reporting here. We deem that if from one side, this does not allow deriving highly confident levels of statistical significance, from the other, the data we collected can indicate meaningful insights for the use case at hand, allowing us to identify criticalities and lessons learned. Questionnaires and Likert items were chosen following the indications derived from focus groups with elders [12], so that they can provide meaningful indications on the entire system as a whole and on single components and functionalities.

### Questionnaire Analysis

No incident or major failure occurred during the pilot. The system operated safely in all the houses for the entire pilot duration. During the study, only one participant from Spain, group ES-HOME, dropped out from the pilot because they did not feel confident with the system. More precisely, they reported that they felt the system was not working correctly. Further investigation identified that the low responsiveness of the robot caused by the limited reliability of the network inside the apartment was responsible for these issues. This also made the CBAC difficult to use. Following the dropout, a total of 24 elders completed the pilot trial.

Another user from the same group, ES-HOME-1, experienced similar technical and connectivity issues. However, they decided to carry on with the study and eventually provided a significantly lower evaluation than the other participants from ES-HOME. In our analysis ES-HOME-1 was thus considered an outlier. The same holds for the four ITA-AL users that, due to their average gap in age and MMSE score (see Sect. 5.1), provided different remarks and evaluations when compared with all the other 19 participants. For that reason, in some analysis, we also evaluate the results separately from the questionnaires as answered by these 5 users (ES-HOME-1 and ITA-AL), labelling them as  OUTLIER when explicitly mentioned.

At the end of either round R1 or R2, participants were requested to fill the questionnaires. Participants who took part in both pilot rounds filled the questionnaires only at the end of R2.

No statistically significant differences were found between the ratings of the Italian and Spanish samples in any question of all questionnaires (U-Mann Whitney test, $\rho >0.05$). This is true both when the Italian group is constituted of ITA-HOME and ITA-AL users or by only ITA-HOME users.

#### Satisfaction Questionnaire

The satisfaction questionnaire aims at evaluating the overall perception of the system. Obtained results are detailed in Table 4 by showing median *M* and interquartile ranges *IQ*. (Table 6, in the Appendix reports the complete answers for all users to questionnaires.) As it can be seen, answers have been particularly positive, which shows how the users appreciated the system and the set of functionalities it provided. This fact is important as users had the possibility to test extensively the system in their own apartments during the weeks of the pilot.

We have also analysed the possible impact of the robot on satisfaction. Statistically significant differences were found in question Q1, “I felt at ease when using the system”, between the group of with-robot and without-robot, with without-robot users who rated the system more positively than the others (the U Mann Withney test showed a $U=35$, $p=0.026$, $median=5$ and 4).

Moreover, the U-Mann Whitney test showed that the users of R2 (both with-robot and without-robot) were significantly more satisfied with the systems than users of R1 ($Q2, U=34.5$, $p=0.042$, $median=5$ and 4; *Q*3, $U=33$, $p=0.029$, $median=1$ and 3). In this respect, R1 has indeed allowed identifying typical technical issues that arouse at home, providing improvement in the connectivity and in automatically managing them. This has allowed getting a more robust system for R2 with a better appreciation by the elders.

#### System Usability Scale (SUS)

The SUS questionnaire was used to evaluate the general system usability. A system is considered to have passed the usability test if achieving a total score above 68 [6]. The SUS score we collected for each participant is reported in Table 5 (lumped results are reported for each of the 10 questions in Table 7, in the Appendix) The results we obtained are, in general, positive and confirming the indication obtained from the satisfaction questionnaire. After experiencing the system for several weeks, and despite the complexity of the proposed system, users reported that they would like to use the system frequently (Q1) and that they felt that the system was not unnecessarily complex and easy to use (Q2-3). However, they also reported how they perceived the system not to be well integrated, with some inconsistencies (Q5-6).

As can be seen in Table 5, the SUS score is highly variable. A minimum of 5 has been assigned by user ES-HOME-1 (the user that experienced connection issues); this is far lower than the next lowest value that is 37.5, and it was provided by a different user from the ES-HOME group interestingly still belonging to R1. A maximum of 90/100 was assigned by two other ES-HOME users.

When evaluating *M* and *IQ* for users who tested the system with-robot and without-robot, we see that the robot reduces system’s usability from 72.5 to 57.5. We remark how by pulling out the 5  OUTLIER users (user ES-HOME-1 and the whole ITA-AL group) the median value increases for without-robot users (from 72.5 to 76.3) and for with-robot users (from 57.5 to 60.0).

**Fig. 9:**
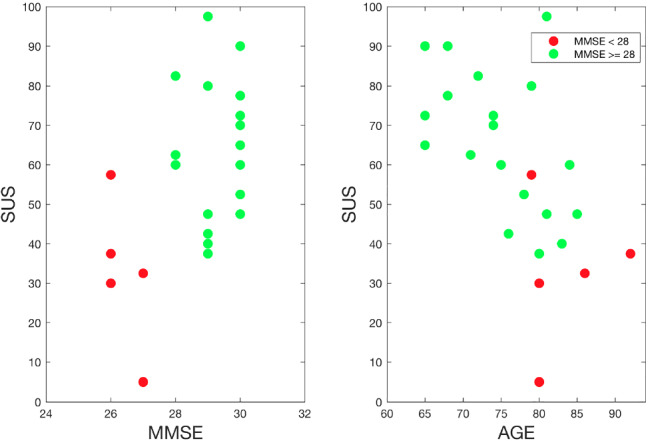
Scatter plots of the SUS score, the MMSE, and age; users with MMSE below 28 are in red while others are in green

Users with low MMSE, as can be seen in Fig. 9, show the lowest SUS score. Indeed, a non-parametric bivariate correlation between MMSE at baseline and total SUS score was computed using the Spearman coefficient. A significant positive correlation was found ($\rho = 0.530$, $p=0.01$). This result confirms that the platform was perceived as more usable by people having the best cognitive function.

The SUS score also decreases with age. Indeed, a non-parametric bivariate correlation between age and total SUS score was computed using the Spearman coefficient. A significant negative correlation was found ($\rho =-0.636$, $p=0.001$). This result confirms the worst results obtained by ITA-AL users that were characterised by a median age of about 10 years older than the users recruited in the ITA-HOME and ES-HOME groups.

Users from ES-HOME with-robot were interested in using the system, and they perceived its utility. However, their main concerns were related to the robot, as expressed by open questions. The dimension of the robot was perceived as too big for their rooms’ dimensions, and the users would have liked to have a more responsive robot.

If we evaluate the median *M* and percentile *IQ* scores obtained separately by the participants to R1 and R2 we can see how the median increases from 47.5 to 65. The removal of  OUTLIER also increases scores both in R1 (from 47.5 to 53.7) and R2 (from 65 to 70).

None of the ITA-AL users reported a positive SUS score without improvement in SUS score between R1 and R2. This confirms the fact that SUS scores observed were correlated with users’ age and MMSE, suggesting how older users found the system more difficult to use.

The median value of the SUS score between R2 and R1 improved of 19 points for ITA-HOME users. Overall, 57% of ITA-HOME users rated the system above the threshold, of which 2 with-robot and 2 without-robot. Among all the users in R2, two of them also belonged to R1 and therefore 80% of the users of R2 reported a SUS above the threshold, with an average value of 75/100.

The small changes performed to improve the system between R1 and R2 significantly improved its system usability score from below threshold to above or close to the threshold. Users perceived the changes in terms of improved usability (a change in the SUS score of about 20 points was obtained both in ITA-HOME and ES-HOME groups).

Note that most of the improvements provided were due to the connection’s unreliability inside the pilot apartments. This shows how, in a complex system like this one, issues identified in single components can lower the overall evaluation, as the user perceives the system as more fragile and, consequently, less useful.

#### System Validation Questionnaire

The System Validation Questionnaire and the *M* and *IQ* values for all questions are reported in Table 8, in the Appendix. This questionnaire received positive answers too since no question has collected a median value less than 3 over 5. This shows again how the users appreciated the functionalities proposed by the system even after a prolonged interaction lasting several weeks, and that they appreciated it both in the without-robot but also, and most importantly, in the with-robot condition.

As a general result, the 79% of the participants stated that they felt comfortable using the system surrounded by family and friends all or most of the time (Q9), 65% of participants positively evaluated the satisfaction with the study regarding the ease of learning all individual functions (Q11), and the 67% of participants considered the system safe (Q3). Answers reported that the users felt safe and comfortable when using the system (Q4 and Q15). In this validation questionnaire, no significant associations between the answer value (1-5) and the robot condition were found. Despite the long duration of the pilot, the users reported that they were comfortable when using the system and appreciated its contribution to the improvement of their life and its adaptability to the spaces they spend their everyday life in (Q1,Q2). This result is particularly meaningful for users with-robot as Giraff-X, despite its size, moved autonomously inside their apartments without being perceived as intrusive.

However, open answers from the participants with-robot shown how users did not perceive the robot as helpful or responsive as expected. They also felt that interacting with the robot through vocal commands was not easy and that the “feedback from the robot should be more informative”. These comments, as well as the slightly lower evaluation provided by with-robot users show how, despite with-robot users appreciated the Giraff-X’s functionalities and its presence in their own houses, there is still considerable margin for improvement to unlock its potential fully.

**Fig. 10:**
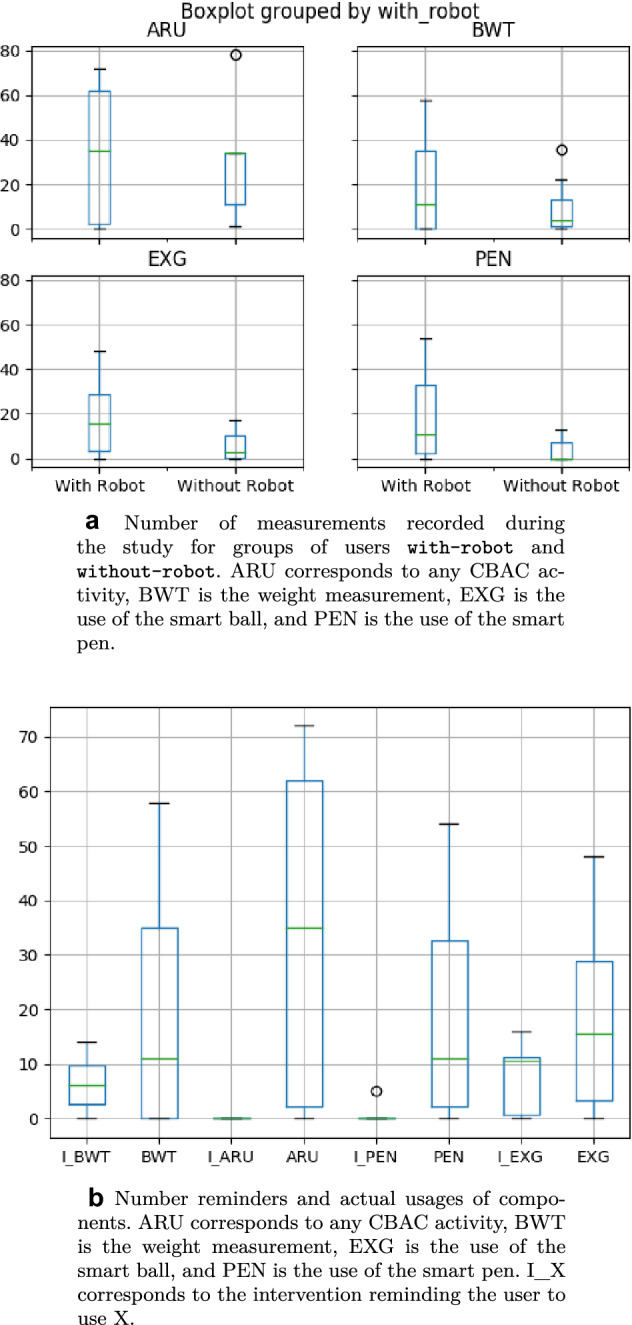
Reminders and usage of the system

### Interaction Between the Components

We also analysed the effect of the interaction between different components. Figure 10a shows the number of measurements corresponding to the use of the CBAC (code ARU, standing for Activity Report for a User), the use of the smart scale (code BWT, standing for Body Weight measuremenT), the use of the smart ball (code EXG, standing for EXerGame), and the use of the smart pen (code PEN) during R2 and regarding all users participating to it. We can see that robot’s presence systematically increases the use of the Smart Objects and the CBAC. Moreover, when analysing the number of interventions delivered to users with-robot compared to the number of actual measurements from the same users (Fig. 10b) we can see that the users with-robot are interacting with the monitoring system much more often than they are reminded to. This suggests that either robot’s physical presence or the few times the robot actually reminded them to use the object was sufficient to encourage users to use the functionalities provided by the system regularly. This could also result from the fact that users that have been equipped with a robot are more engaged with the study, and therefore more likely to use the system. Specifically, all with-robot older adults (except  OUTLIER ones) used most of the functionalities of the system for the entire duration of the pilot; at the same time, several without-robot older adults only used the platform during the initial days of the pilot and stopped doing so afterwards, eventually losing interests in it. Furthermore, not only the robot encouraged the users to use the smart objects regularly but also informed them in case incorrect values were measured or when the users failed to comply with the agreed protocol (i.e., to measure their weight only in the morning after getting up), suggesting them to repeat the measurement. It emerges how Giraff-X played a role in how much the users used the system, which strengthens the hypothesis that a robotic assistant associated with a monitoring system could be preferable to a monitoring system alone [14].

Overall, most older adults participants valued the call for help scenario as an extremely useful tool in an emergency, with the great majority of them feeling remote assistance would be helpful and all of them reporting that it would make them “feel calmer” knowing the robot can facilitate them getting help from their caregiver through telepresence. Again, these numbers are encouraging and suggest an overall satisfaction with this scenario. Almost every participant agreed that the robot manages emergency situations in a way that makes them feel comfortable, safe, and is effective. This is also confirmed by the answers to open questions.

## Discussion

To the best of our knowledge, our study is the first to tackle the challenge of a heterogeneous, highly integrated, autonomous system concurrently deployed in multiple domestic installations for a long time to provide stimulation, monitoring, and assistance to older adults. The extensive on-the-field experimental campaign we described in this work can contribute to a better understanding, from a long-term perspective, of the acceptability and feasibility of SARs integrated with AAL platforms. Specifically, the data we collected during 300 weeks of usage in uncontrolled environments allowed us to derive meaningful practical insights. In the following sections we discuss about the lessons we learned, the limitations that we encountered, and the open questions still calling for future improvements.

### Lessons Learned

In general, participants reported feeling safe and comfortable with the system and believed that instructions were clear and the system was generally easy to use. The digital divide has been shown to have less impact than expected, as elderly users can use technology much easier than they believe [12]. Nevertheless, some users, especially those with little familiarity with technology, reported that too many new notions had to be learned at installation time and needed support. For this reason, training was conducted at a slow pace (sometimes over multiple sessions) to avoid flooding the elder with too many notions in a short time. Interestingly, the most of the people who felt to be more confident in using the system autonomously were those that received the platform without the robot, a component that, indeed, adds complexity to the platform. Such a result confirms the importance of training, from simple basic functionalities to more complex ones. Still, some form of digital divide was observed analysing the answers by age as older people tended to rate the system as more difficult to use and less compliant, while younger people were more enthusiastic and proficient in using it.

Giraff-X played a crucial role in those platform deployments that included it. Almost every participant appreciated the Call for Help scenario, reporting that it made them feel comfortable and safe. In the pilot, no elder needed help, but some elders tested the functionality for exploring it and even for fun (for example, to see how the robot would approach them to ask if they needed help, to which they would answer “no thank you”). Seeing the robot responsive and willing to help turned out to boost the confidence towards the system. Moreover, all the participants were proud to show their “mechanical assistance” to their friends and relatives; this recalls the boost of mechanical automata that developed throughout the prize to Human ingenious [57]. Moreover, such an intervention modality is much broader than that implemented by most AAL systems that aimed only to detect falls (e.g.,[3]): indeed elders may need help not only because of falls but also for many other issues, and the possibility of getting an emergency contact with caregivers without the need to wear any device (e.g., smartwatch or an alarm button) is seen as a step forward.

A covert advantage of the robot’s presence was an increase in the use of the other components, particularly those related to monitoring functionalities. Interestingly, the robot did play a role in how much the users used the monitoring system, which strengthens the hypothesis that a SAR associated with a monitoring system is preferable to a monitoring system alone [14]. Figure 10a shows the boxplot corresponding to the number of times elders with-robot and without-robot used the smart-ball and the smart-pen. These plots demonstrate the great impact of a mobile robot to encourage users to actively use smart objects (and consequently to gather health monitoring data). This consideration is more evident if we do not consider in the analysis the  OUTLIER users that, for different reasons, did not exploit the system in its entirety. Removing them shows how most of the users with-robot used smart objects more frequently, while several users without-robot used them only in the first days of the pilot and then neglected them. It must be stressed that, for without-robot users, the system was still able to send notifications and reminders to the user through the CBAC tablet [62]. However, the embodiment of the robot [14] resulted in a more effective strategy.

Similar remarks could be made for other functionalities provided by the system, as the one to foster socialisation through the use of the CBAC. Users with-robot were more engaged in performing social activities with peers on the CBAC, and forming social bonds that they kept after the pilot study [39].

On the other side, the general perception was that the robot had an excessive size and a smaller one would have been appreciated. Some indications to improve reactivity and speed were collected as well. Indeed, speed was limited to provide safe navigation and to make the robot’s movements more predictable. Nevertheless, the lack of direct control of the robot was not appreciated by some users. The opinion that a richer conversation with the robot could make it more acceptable was also reported. Finally, some users who had the robot in R1 asked to continue using of the platform in R2 because they perceived the robot’s utility.

The long duration of the experimental campaign and positive remarks to questionnaires provide strong evidence that a system like the one presented here could be effectively deployed in the long term in an AAL setting. Although that the robot was perceived as ‘big’ by users, and could be perceived as an invasive presence inside the user house, only one user dropped out from the pilot study; all the other ones completed the evaluation. This shows how, despite SARs are still a new technology with several limitations and limited autonomy, their daily use is possible even nowadays.

The difference in evaluation between users of R1 and R2 shows the importance of such testing in real deployment conditions. Even small changes that improve and ‘polish’ the system are important, as minor flaws can reduce the overall evaluation of the system. However, such improvements and fixes could not be envisioned in advance but are the results of constant feedback received directly from end-users. Note that we had already experienced long-term deployment of the system in pre-pilots studies performed with expert users. Some issues that emerged in these pre-pilots had been already fixed before R1 [40].

Albeit limited, the experience of the three users who tested the system in R1+R2 with-robot is of interest, as they were able to assess the capabilities of the robot for more than 20 weeks. Those users reported mixed feedback: they overall appreciated the system and its functionalities but reported that having a more ‘polished’ integration between components could be beneficial.

Results show that the use of the whole platform, and the CBAC in particular, was vastly increased after the COVID-19 pandemic outbreak. This was particularly evident for the ITA-HOME group, where a strict national lockdown was in place for the last three weeks of R2 (and with early restrictive measures already in place in the two previous weeks). The forced reclusion acted as an additional incentive for users to seek support through the platform. Interestingly, one of the ITA-HOME users at the end of the pilot period, with the COVID-19 national lockdown still in place, requested to keep the system and the robot active for two additional months. She then provided positive feedback reporting that the system and the robot’s presence “was of great support during the difficult time”. Indeed, albeit limited, the experience of R2 took place during a national lockdown and shows how AAL frameworks and SARs could be of help to support older adults from the consequence of the COVID-19 pandemic, which affects frail and pre-frail older adults users particularly [16].

Smart objects needed maintenance from users, as they needed to be recharged. This did not always happen, and some software functions have been introduced to allow the VC to remind elders to recharge the objects after using them. The robot was a powerful tool to do so, and users with-robot used smart objects more than those without-robot.

In general, open answers reported by users shown how they liked very much digital neuropsychological tests, which open the door for a possible massive deployment of screening tests. In a recent study, we assessed the clinical validity of these tests. The study also highlighted the potentiality of providing additional and finer quantitative indexes to clinicians [38]. Users also liked the digitalised tests and answering spot questions while doing their daily activities. In general, they positively evaluated scenarios where they had the chance to interact with the robot.

When the robot needed help, it asked the user to contact a technician or move back the robot manually to the docking station [43]. Users performed such ‘assistive’ functionality to the robot without signalling it as a robot limitation or reporting it as an unwanted or obtrusive system requirement. Some users even signalled that they were happy to trigger the robot to move in the apartment and to support it during its tasks. This shows how a synergy could be created in the long term between older adults and SARs.

From a technical point of view, such a system is highly dependent on network connectivity, not only in terms of bandwidth but also in availability and latency. Although some improvement was carried out between R1 and R2 in managing this issue, for some elders (e.g., ES-HOME-1), multiple network failures made the system little usable.

### Limitations and Open Questions

The main result of this study is to provide strong empirical evidence that Socially Assistive Robots could be successfully used for long-term assistance of older adults. The with-robot configuration improved the effectiveness of the whole system in assisting the elder. As a result, participants with-robot used the system’s assistive functionalities for more time, also allowing the system to be more efficient in collecting meaningful monitoring data about them.

However, as a negative side effect, the robot’s presence lowered the evaluation of the system. Consequently, while our study showed the feasibility of long-term assistance and monitoring of SAR-based systems, their acceptability is not entirely ascertained. Future works should investigate how to improve interactions between older adults and SARs by focusing on the effects of the long-term interaction (LT-HRI) between them; after an initial ‘novelty effect’, the user may become tired of the robot and consequently require different interaction methods. Despite this, almost all users (except one who dropped out) completed the study, which should be seen as a positive indication of such a scenario.

Our work presented a proof of concept of how different functionalities as monitoring, assistance, and stimulation could be integrated under a unifying framework. However, each one of these functionalities would require a deeper analysis. In particular, while we have shown how it is possible to collect monitoring data through distributed sources, the development of a clinically effective monitoring platform requires a more extensive analysis in terms of sample size and pilot duration in order to allow a longitudinal analysis, and to perform controlled tests on selected variables of interest. A similar remark could be made to investigate the long-term effect on social stimulation using the CBAC and physical monitoring through smart objects.

Another relevant limitation is that we observed a correlation between age, MMSE, and the evaluation of the system: our proposed system was more effective with younger and healthier older adults than with older ones. In the long term, SAR-based systems should support not only healthy and ‘young’ older adults but all types of subjects, as health conditions may change with time. Therefore, future works should investigate which are the limitations of the long-term deployment and interactions of SAR with older adults at different stages of MCI towards their full acceptance.

Answers to questionnaires shown how the presence of (even minor) inconsistencies in the integrated system widely affects its overall evaluation; improvements between R1 and R2 increase the scoring, but questions related to integration received the lowest scores.

The VC’s main role was to *functionally* manage the entire system by orchestrating and executing scenarios. However, such a component has a covert yet essential role in managing the entire platform from a technical standpoint, as it is in charge of identifying, reacting, and adapting the system to unexpected events that could be due to malfunctioning or external causes. When evaluating AAL and SAR-based frameworks, these self-management functionalities are often neglected. Real-world deployment of these systems requires a deeper analysis of these issues to be robust while also allowing proactivity in early identification and solving of possible causes of failure. As more extended deployments of such systems naturally increase the risks of system failures, the monitoring role of an AAL system orchestrator, as the VC is, should be not only focused on the user but also on the system itself.

Finally, another limitation is in the limited sample size of users who participated to our experimental campaign. Larger and longer experiments should be performed to collect more significant data. Such longer experimental campaigns can perform longitudinal data analysis and observe how such an HRI evolves over time. In that cases, the introduction of modifications in the robot behaviour (e.g., by trying to introduce an emphatic behaviour of the robot, adapting it to the users’ status) could be beneficial.

## Conclusions

This paper tackled the open problem of integrating Ambient Assisted Living platforms and Socially Assistive Robots for at-home monitoring, stimulation, and assistance of older users. We did this by devising the MoveCare platform, a system that integrates and orchestrates a set of specific components to provide complex and tailored monitoring, stimulation, and assisting functionalities at home to the elders at risk of frailty in their own houses. We focused and reported on the long-term deployment of our system, analysing the feasibility and acceptability of its complex functionalities when deployed in real users’ homes. By comparing the results obtained from pilots with the whole system, and those without the mobile robot, we provided evidence that integration of AAL and SARs is beneficial for monitoring, stimulation, and assistance. On the one hand, the inclusion of the service robot improved the set of capabilities provided by the system and intensified the usage of the whole platform; on the other, it decreased its acceptability.

These mixed findings suggest that the use of SARs integrated with AAL platforms do enable several functionalities that are desired and needed by older adults and caregivers. At the same time, there is still a technological gap in SARs that needs to be assessed through extensive evaluations to unlock their full potential. New platforms should provide better performance in terms of responsiveness and by naturally interacting with users through extensive dialogues.

Our results positively suggest that, in the future, ambient assisted living frameworks integrated with socially assistive robots can be successfully used to monitor the cognitive and physical state of older adults at home and provide assistance in their everyday lives.

## Figures and Tables

**Fig. 11:**
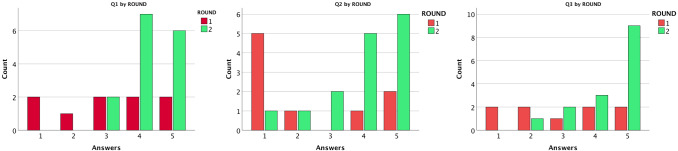
Distribution of the answers obtained in R1 R2 for System Satisfaction Questionnaire

**Fig. 12:**
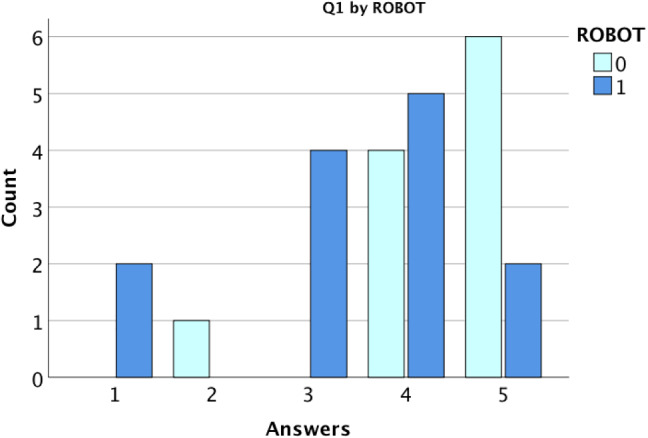
Distributions of the answers to the question Q1 given by participants testing the system with (light blue) or without robot (blue). Please notice that this question is positive and thus the answer 5 is the best possible answer

**Table 6:**
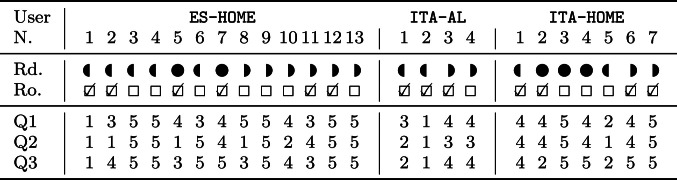
Individual answers to the Satisfaction Questionnaire by all users

; ; ; ; ;

**Table 7:**
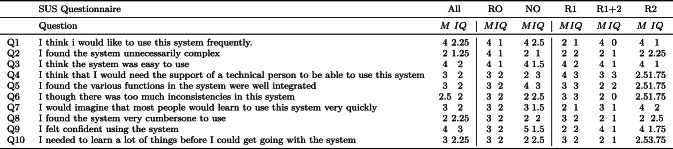
SUS Qustionnaire [6]. Note that the SUS questionnaire the odd numbered questions express positive attitudes, while the even ones express negative attitudes. RO is with-robot and NO is without-robot

## Data Availability

The data that support the findings of this study are provided as supplementary material. Full answers to questionnaires are not publicly available due to restrictions, as they contain information that could compromise the privacy of research participants. Anonymised versions of such data are available upon request made to the corresponding author, N.A.B. Full data acquired from the platform and that document the platform usage by users are not available due to privacy issues of the participants, as they contain details of the daily activities performed by users. Further details upon the methods used for the collection of these data and their content are available upon request made to the corresponding author. All data are available to the corresponding author for further inquiries.

## Appendix

### SUS

See Table 7.

### System Validation Questionnaire

See Table 8.

**Table 8:** System Validation Questionnaire. *M* is Median, *IQ* is InterQuartile range, *N* is the number of pilot participants who answered to that question

	System Validation Questionnaire		
	Question	*M*	*IQ*
Q1	Rate your satisfaction with MoveCare in relation to its contribution to the improvement of your everyday life.	4	1.25
Q2	Rate your satisfaction with MoveCare in relation to the adaptability in the spaces you spend your everyday life.	4	1.25
Q3	Rate your satisfaction with MoveCare in relation to how safe it is.	4	1.25
Q4	Rate your satisfaction with MoveCare in relation to the degree to which the system meets your needs.	3	2
Q5	Rate your satisfaction with MoveCare in relation to the responsiveness of the system to your inputs.	3	2
Q6	Rate your satisfaction with MoveCare in relation to the reliability of the system.	3	2
Q7	I will feel more confident when using MoveCare.	4	1.5
Q8	I will feel more connected with the external world when using MoveCare.	4	3
Q9	I will feel at ease when using MoveCare around friends and family.	4	1
Q10	MoveCare allowed me to establish new social connections with the external world.	3	3
Q11	Rate your satisfaction with MoveCare in relation to the ease of learning all individual functions.	4	3
Q12	Rate your satisfaction with MoveCare in relation to the ease of interacting with the system.	4	1.25
Q13	Rate your satisfaction with MoveCare in relation to the ease of use.	4	1.25
Q14	I will feel more autonomous when using MoveCare.	3	3
Q15	I will feel comfortable when using MoveCare.	4	2.25
Q16	I will feel like to have control over the system when using MoveCare.	3	2.5

### Components and hardware

See Table 9.

**Table 9:** Detailed list of all the components that compose the MoveCare framework and of their use within the system

Component	Use
Giraff-X	Socially Assistitive Robot
Giraff	Main robot platform; previously used as a telepresence robot. Redeveloped for the project running ROS and as autonomous.
(top) RBGD camera	Robot’s sensor. Used for obstacle avoidance, user detection, navigation.
(lower)RBGD camera	Robot’s sensor. Used for people detection and as main robot microphone.
Hokuyo URG lidar	Robot’s sensor. Used for SLAM, navigation.
nVidia Jetson	Robot’s component. Additional computation for the robot (user detection).
RFID reader	Robot’s sensor. Used for Search for Lost Objects scenario.
Docking station	Placed inside the house in an easily accessible position, it is the resting place for the robot when charging. When the robot has no active intervention to perform, it stays connected to the docking station.
RFID tags	Placed on a set of objects (keys, glasses, remote controller, wallet), the robot uses them to track the object upon user request.

IoT Network	Environmental monitoring
PIR sensors	User presence inside the house/room. One/two PIRs were used to cover each room entirely.
Smart plug	Connected to the main TV, monitors its usage (on/off).
Door sensor	Connected to the main door, monitors if the user enters/exits the house.
Thin accelerometer	One placed under the bed mattress, one under the sofa. Used to monitor sleeping/resting behaviour.
Microphones	Two or more for each flat, so the entire flat is covered by at least one. Employed to detect user’s commands like requests for help, or commands to the robot (e.g., ‘Go away!’).
Master switch	Turns off/on the system.
Concentrator	Central node for all environmental sensors and smart objects, it provides internet, BLE, and ZigBee connectivity, stores the map of the environment (as acquired by the robot) and the setup of the system. It has a GPRS card to make phone calls to caregivers in case of an emergency.
Router	Connected with the concentrator via Ethernet, it gives WiFi connection to all components and to the robot.

Smart Objects	Advanced monitoring functionalities
Smart ball	Anti-stress plastic ball with sensors inside, it measures the maximum grip strength and could be used to play some ad-hoc exergame or as a standalone object.
Smart pen	An ink-pen with some sensors embedded that are used to measure strength and tremor of the users while writing on paper.
Sensorized insoles	Placed inside the users’ shoes and used to monitor outdoor activity and gait.
Bluetooth scale	To monitor the user weight.
Charging stations	3D-printed support for charging wireless the smart ball and insoles, and wired charge of the smart pen. Usually, placed close to the television.

CBAC	Social, cognitive, and physical support with tailored interventions
Tablet	Main device for using the CBAC, it runs a dedicated App with all the MoveCare activities
Intel NUC computer	Main component of the TV Set-top box kit. Connected to the main TV it provides access to the CBAC from the TV and runs CBAC and CBAC exergames.
Webcam	Part of the TV set-top box kit. It is placed on top of the TV and is used to do videocalls during CBAC activities made with the TV set-top box.
air-mouse	Part of the TV set-top box kit. Remote controller with an embedded accelerometer that is used to control the CBAC on the TV set-top box.
Balance Board	Part of the TV set-top box kit. It is used as a controller to play with the exergames on the CBAC on the TV set-top box. It measures data about user balance and gait.

### Description of the MoveCare Scenarios

See Table 10.

**Table 10:** Detailed description of the MoveCare Scenarios

Scenario	Description	Changes in without-robot
Call for Help	The user, in case of an emergency, can ask for help through the distributed microphones; the message is forwarded to the robot, who tries to locate the user; when the user is found, the robot asks for a confirmation of the emergency situation. If the situation is confirmed (or if there is no answer from the user and a timeout expires), the IoT concentrator calls the user’s caregivers through a phone call. When a caregiver answers to the call and decides to handle the emergency situation, a link is sent to them to access, upon authentication, a teleoperation session on the robot. From a web interface, the caregivers can teleoperate the robot. The user can refuse the teleoperation session, or close it, by pressing on the red button on the robot or by voice commands through the microphones. When the emergency situation is closed, a report is created.	After the user asks for help, the IoT concentrator immediately calls the caregiver on his/her phone until a caregiver accepts the emergency. After a caregiver do so, the scenario is closed.
Body Weight Measurement	Users are expected to weight themselves regularly. After a few days where no data are collected, the robot makes an intervention and asks the user to do a weight measurement. After the measure is received, the robot say a ‘thank you’ message to the user.	If no data are collected after a certain amount of time, a notification to perform a weight measurement is shown to the user on the CBAC (tablet or TV set-top box).
Neuropsycho- logical Tests	The robot approaches the user and asks them to do a cognitive test on the tablet. When the user opens the tablet, a wait page is shown. The robot then re-identifies the user and, when found, moves close to them. The robot asks the user if they is ready. If so, it triggers the start of the cognitive tests that are performed on the tablet. The robot interacts with the user during the execution by mimicking the behaviour of a caregiver during the execution of such tests (e.g., by giving explanation). When the scenario is completed, the robot thanks the user.	The scenario is not performed in the without-robot setting.
Spot Questions	The robot starts and intervention and approaches the user. The robot asks the user if they have some time to answer a question. If so, the robot asks a question to the user; questions have been developed to assess different cognitive abilities (e.g., ‘what day of the week is today?’).	The scenario is not performed in the without-robot setting.
Grip Force Measurements	The user should do an exergame with the smart ball regularly; if data are missing, the robot performs an intervention by asking the user to either play with the smart ball exergames or by checking the battery level of the smart ball.	If no data are collected after a certain amount of time, a notification to perform a smart ball exergames is shown to the user on the CBAC.
Use of Smart Objects	The user should use the smart pen or the smart ball (as a standalone object) regularly; if data are missing, the robot performs an intervention by asking the user to use a smart objects or to check their battery level.	If no data are collected after a certain amount of time, a notification to use the smart object is shown to the user on the CBAC.
Gentle Exercises	Users should do a gentle exercise session regularly; if no sessions are recorded for a given user, the robot performs an intervention by reminding to the user to use the Gentle Exercise activity.	A notification to do gentle exercise is shown on the CBAC . Also, the activity is prioritised in the CBAC interface.
Balance Exergames	Users should play with balance exergames regularly; if no sessions are recorded for a given user, the robot performs an intervention by reminding to the user to use them.	A notification to do exergames is shown on the CBAC. Also, the activity is prioritised in the CBAC interface.
Outdoor Suggestions	Users should do outdoor activity sessions regularly; if such sessions are not recorded with the insoles, the robot performs an intervention to remind the user to do such activities with the insoles, as well as to check those suggested on the CBAC.	A notification to do outdoor activities is shown on the CBAC. Also, the list of suggested activities is prioritised in the CBAC interface.
Finding Lost Objects	The user, on the CBAC, asks the robot to locate one of the RFID tagged objects. The robot searches for the object inside the house. At the location where the object is found, the robot reports to the user by saying ’I found the object’. Otherwise, the robot signals that the object was not found.	The scenario is not performed in the without-robot setting.

## Footnotes

1 http://www.aal-europe.eu/.

2 https://www.pharaon.eu/.

3 https://www.smart-bear.eu/.

4 http://www.movecare-project.eu.

5 http://wiki.ros.org/navigation.

6 https://www.acapela-group.com/.

7 https://mqtt.org/.

8 https://feetme.fr/en.

9 https://www.korian.it/.

10 The MMSE [19] is commonly used to evaluate cognition; a score above 26 indicates normal cognitive abilities.

11 https://www.anteas.org/.

12 https://www.policlinico.mi.it/.
